# Supplementary material for: Factors associated with calendar literacy and last menstrual period (LMP) recall: a prospective programmatic implication to maternal health in Bangladesh
Source: BMJ Open. 2020 Dec 13;10(12):e036994. doi: 10.1136/bmjopen-2020-036994 (PMC7737077; doi:10.1136/bmjopen-2020-036994)
Supplement: Supplementary data [file bmjopen-2020-036994supp001.pdf]

Supplementary Table 1 Association between calendar literacy and socio-demographic characteristics of the study participants

Traits	Calendar literacy		P-value
	Literate 1730 (%)	Illiterate 1001 (%)	
**Age in years**			
≤19	362(59.44)	247 (40.56)	<0.01
20-29	1135(67.04)	558 (32.96)	
≥30	233(54.31)	196 (45.69)	
**Completed years of schooling**			
<5	52 (9.61)	489 (90.39)	<0.01
5-7	437 (54.90)	359 (45.10)	
≥ 8	1241 (89.02)	153 (10.98)	
**Residence**			
Rural	1,029 (56.66)	787 (43.34)	<0.01
Urban	701 (76.61)	214 (23.39)	
**Employment status of the participant**			
Unemployed	1610 (62.33)	973 (37.67)	<0.01
Employed*1	120 (81.08)	28 (18.92)	
**Gravida**			
1	751 (72.35)	287 (27.65)	<0.01
≥2	979 (57.83)	714 (42.17)	
**Wealth index**			
Poorer	192(35.10)	355(64.90)	<0.01
Poor	295(54.03)	251(45.97)	
Middle	333(60.99)	213(39.01)	
Rich	423(77.47)	123(22.53)	
Richest	487(89.19)	59(10.81)	
**Availability of calendar at home**			
Yes	1244(78.04)	350(21.96)	<0.01
No	486(42.74)	651(57.26)	

**Purpose of using calendar*2**			
To calculate the days and dates	1085(90.12)	119(9.88)	
To track the dates of menstrual period	241(89.93)	27(10.07)	
To remember the special/festival day (Hat day/Eid/Puja/selling certain goods)	342(93.96)	22(6.04)	<0.01
To remember the date of tuition fee of children	77(90.59)	8(9.41)	
To remember the loan installment day	57(93.44)	4(6.56)	
Others*3	36(92.31)	3(7.69)	
*1 refers Service/Business/Handicraft/Agriculture/Farm/Fishing, and Day labor, etc.			
*2 refers multiple responses			
*3 refers child's date of birth, calculate lunar date, etc.			

Supplementary Table 2 Results from logistics regression on calendar literacy

Covariates	cOR (95% CI lower to upper)	aOR	(95% CI lower to upper)	P value
**Constant**		0.05	(0.03 to 0.08)	<0.01*
**Age in years**				
≤19 years	1.23 (0.96 to 1.58)	0.95	(0.61 to 1.47)	0.81
20 to 29 years	1.71 (1.38 to 2.12)	1.20	(0.84 to 1.69)	0.31
≥ 30 years	1.0	1.0		
**Completed years of schooling**				
<5	1.0	1.0		
5-7	11.4 (8.33 to 15.73)	8.35	(5.94 to 11.73)	<0.01*
≥8	76.27 (54.75 to 106.26)	34.46	(23.96 to 49.57)	<0.01*
**Residence**				
Rural	1.0	1.0		
Urban	2.51 (2.09 to 2.99)	1.20	(0.85 to 1.69)	0.30

**Employment status of the participant**				
Unemployed	1.0	1.0		
Employed*	2.59 (1.70 to 3.94)	1.83	(0.99 to 3.38)	0.05
**Gravida**				
1	1.91 (1.61 to 2.25)	1.21	(0.91 to 1.60)	0.18
≥2	1.0	1.0		
**Wealth index**				
Poorer	1.0	1.0		
Poor	2.17 (1.70 to 2.77)	1.71	(1.25 to 2.34)	<0.01*
Middle	2.89 (2.26 to 3.69)	1.28	(0.92 to 1.77)	0.139
Rich	6.36 (4.87 to 8.30)	2.75	(1.83 to 4.11)	<0.01*
Richest	15.26 (11.06 to 21.06)	3.15	(1.88 to 5.29)	<0.01*
**Availability of calendar at home**				
No	1.0	1.0		
Yes	4.76 (4.03 to 5.63)	0.65	(0.48 to 0.88)	<0.01*
**Purpose of using calendar**				
To calculate the days and dates/ installation day	12.47 (10.06 to 15.46)	6.41	(4.57 to 8.98)	<0.01*
To track the dates of menstrual period	5.84 (3.89 to 8.76)	1.08	(0.66 to 1.76)	0.76

Significant p value ≤0.05.
aOR, adjusted OR; cOR, crude OR.
CI – Confidence interval
*refers Service/Business/Handicraft/Agriculture/Farm/Fishing, and Day labor, etc.
Model adjusted for variables such as woman's age, completed years of schooling, residence, employment status, gravida, wealth index, availability of calendar at home, purpose of using calendar
