# Supplementary material for: Factors associated with calendar literacy and last menstrual period (LMP) recall: a prospective programmatic implication to maternal health in Bangladesh
Source: BMJ Open. 2020 Dec 13;10(12):e036994. doi: 10.1136/bmjopen-2020-036994 (PMC7737077; doi:10.1136/bmjopen-2020-036994)
Supplement: Author's manuscript [file bmjopen-2020-036994.draft_revisions.pdf]

BMJ Open

BMJ Open is committed to open peer review. As part of this commitment we make the peer review history of every article we publish publicly available.

When an article is published we post the peer reviewers' comments and the authors' responses online. We also post the versions of the paper that were used during peer review. These are the versions that the peer review comments apply to.

The versions of the paper that follow are the versions that were submitted during the peer review process. They are not the versions of record or the final published versions. They should not be cited or distributed as the published version of this manuscript.

BMJ Open is an open access journal and the full, final, typeset and author-corrected version of record of the manuscript is available on our site with no access controls, subscription charges or pay-per-view fees (http://bmjopen.bmj.com).

If you have any questions on BMJ Open's open peer review process please email info.bmjopen@bmj.com

BMJ Open

Factors associated with calendar literacy and Last Menstrual Period (LMP) recall: a prospective programmatic implication to maternal health in Bangladesh

Journal:	*BMJ Open*
Manuscript ID	bmjopen-2020-036994
Article Type:	Original research
Date Submitted by the Author:	15-Jan-2020

Complete List of Authors:	Sarker, Bidhan Krishna; International Centre for Diarrhoeal Disease Research Bangladesh, Maternal and Child Health Division<br>Rahman, Musfikur; International Centre for Diarrhoeal Disease Research Bangladesh, Maternal and Child Health Divison<br>Rahman, Tanjina ; International Centre for Diarrhoeal Disease Research Bangladesh, Maternal and Child Health Division<br>Rahman, Tawhidur; International Centre for Diarrhoeal Disease Research Bangladesh, Maternal and Child Health Division<br>Rahman, Fariya; International Centre for Diarrhoeal Disease Research Bangladesh, Maternal and Child Health Division<br>Khalil, Jubaida; International Centre for Diarrhoeal Disease Research Bangladesh, Maternal and Child Health Division<br>Hasan, Mehedi; International Centre for Diarrhoeal Disease Research Bangladesh, Maternal and Child Health Division<br>Mahfuz, Sadia; University of Western Sydney<br>Ahmmed, Faisal; International Centre for Diarrhoeal Disease Research Bangladesh, Infectious Diseases Division<br>Miah, Muhammad Salim; Shahjalal University of Science and Technology, Department of Anthropology<br>Ahmed , Anisuddin; International Centre for Diarrhoeal Disease Research Bangladesh<br>Mitra, Dipak; North South University, Public Health<br>Mridha, Malay; BRAC University James P Grant School of Public Health<br>Rahman, Anisur ; International Centre for Diarrhoeal Disease Research, Maternal and Child Health Division

Keywords:	PUBLIC HEALTH, Health policy < HEALTH SERVICES ADMINISTRATION & MANAGEMENT, OBSTETRICS, GYNAECOLOGY, EPIDEMIOLOGY, STATISTICS & RESEARCH METHODS

SCHOLARONE™
Manuscripts

I, the Submitting Author has the right to grant and does grant on behalf of all authors of the Work (as defined in the below author licence), an exclusive licence and/or a non-exclusive licence for contributions from authors who are: i) UK Crown employees; ii) where BMJ has agreed a CC-BY licence shall apply, and/or iii) in accordance with the terms applicable for US Federal Government officers or employees acting as part of their official duties; on a worldwide, perpetual, irrevocable, royalty-free basis to BMJ Publishing Group Ltd ("BMJ") its licensees and where the relevant Journal is co-owned by BMJ to the co-owners of the Journal, to publish the Work in this journal and any other BMJ products and to exploit all rights, as set out in our licence.

The Submitting Author accepts and understands that any supply made under these terms is made by BMJ to the Submitting Author unless you are acting as an employee on behalf of your employer or a postgraduate student of an affiliated institution which is paying any applicable article publishing charge ("APC") for Open Access articles. Where the Submitting Author wishes to make the Work available on an Open Access basis (and intends to pay the relevant APC), the terms of reuse of such Open Access shall be governed by a Creative Commons licence – details of these licences and which Creative Commons licence will apply to this Work are set out in our licence referred to above.

Other than as permitted in any relevant BMJ Author's Self Archiving Policies, I confirm this Work has not been accepted for publication elsewhere, is not being considered for publication elsewhere and does not duplicate material already published. I confirm all authors consent to publication of this Work and authorise the granting of this licence.

For peer review only

Full Title

Factors associated with calendar literacy and Last Menstrual Period (LMP) recall: a prospective programmatic implication to maternal health in Bangladesh

Short title

Associated factors related to calendar literacy and LMP recall

# Corresponding author

Bidhan Krishna Sarker[1], Assistant Scientist, Maternal and Child Health Division, icddr,b. 68, Shaheed Tajuddin Ahmed Sarani, Mohakhali, Dhaka-1212, GPO Box-1000, Dhaka, Bangladesh, E-mail: bidhan@icddrb.org; bidhanicddrb@gmail.com

Authors

Bidhan Krishna Sarker[1], Musfikur Rahman[1], Tanjina Rahman[1], Tawhidur Rahman[1], Fariya Rahman[1], Jubaida Jahan Khalil[1], Mehedi Hasan[1], Sadia Nishat Mahfuz[2], Faisal Ahmmed[3], Muhammad Salim Miah[4], Anisuddin Ahmed[1], Dipak Kumar Mitra[5], Malay Kanti Mridha[6], Anisur Rahman[1]

Authors and Affiliations

[1]Maternal and Child Health Division, icddr,b. 68, Shaheed Tajuddin Ahmed Sarani, Mohakhali, Dhaka-1212, GPO Box-1000, Dhaka, Bangladesh

[2]MS student, University of Western Sydney, Australia

[3]Infectious Diseases Division, icddr,b. 68, Shaheed Tajuddin Ahmed Sarani, Mohakhali, Dhaka-1212, GPO Box-1000, Dhaka, Bangladesh

[4]Lecturer, Department of Anthropology, Shahjalal University of Science & Technology, Sylhet, Bangladesh

[5] Professor and Chair, Department of Public Health, North South University, Dhaka, Bangladesh

[6]Associate Professor and Director of Centre of Excellence for Non-Communicable Diseases and Nutrition, James P Grant school of Public Health, BRAC University, Dhaka, Bangladesh

For peer review only - http://bmjopen.bmj.com/site/about/guidelines.xhtml

Abstract

Objective: This paper explored the factors associated with calendar literacy and Last Menstrual Period (LMP) recall.

Method: We conducted a cross-sectional study in two rural areas and one urban area from three Northern districts of Bangladesh. A semi-structured survey tool was used to collect data from August to November, 2016. The primary outcome variables were calendar literacy and LMP recall. We enrolled women who had delivery outcomes of live birth in last one year prior to conducting interviews. In each study area, around 900 women were interviewed and finally, we completed 2731 interviews from the 3 study areas.

Results: Majority women (77%) from urban area were capable of using any type of calendar however; it was 57% in rural areas. Around 61% women were able to use English and 16% used Bengali calendar in both areas. After adjusting covariates, the odds ratio of calendar literate women with 8 or more years of schooling was 34.5 times (adj. OR 34.46, 95% CI: 23.96, 49.57) higher than women who did not pass primary. The women who belong to the top two wealth quintiles (i.e. rich adj. OR 2.75, 95% CI: 1.83, 4.11 and richest adj. OR 3.15, 95% CI: 1.88, 5.29) were 2.75 and 3.15 times respectively more calendar literate than poor women. Overall 53% women could recall their recent LMP. The women who were calendar literate, about 60% of them could recall their recent LMPs. The odds ratio of recalling LMP for calendar literate women was 1.59 times (adj. OR 1.59, 95% CI: 1.28, 1.98) higher than calendar illiterate women.

Conclusion: LMP recall is associated with calendar literacy. Calendar literacy is an opportunity to introduce a simple tool like a calendar through existing maternal health system to help women to track their LMPs regularly in improving maternal health outcomes in Bangladesh.

For peer review only - http://bmjopen.bmj.com/site/about/guidelines.xhtml

Strength and Limitation of this Study

1. This is the 1st large-scale study in Bangladesh reporting the prevalence of last menstrual period (LMP) recall with both urban and rural settings.
2. The study relied on the self-reported data and also cross-checked the actuality of the data and the validity of the data rechecked through re-interview
3. We took into account of both government and non-government health service catchment areas maintaining similar population coverage and statistical rigor
4. Upon its limitation, the study cannot demonstrate a nationwide prevalence of LMP recall.

For peer review only

For peer review only - http://bmjopen.bmj.com/site/about/guidelines.xhtml

Introduction

About 830 women die from pregnancy or childbirth-related complications around the world every day. Almost all of these deaths occur in low-resource settings, and most of them are preventable,[1]. The south Asia region alone accounts for approximately one-third of the global maternal and child deaths annually,[2]. Globally new target set by Sustainable Development Goals (SDG) for maternal mortality ratio is to reduce less than 70 per 100,000 live births by 2030,[1].

Pregnancy is an important part of women's life. It is very crucial for women to have awareness about their own health,[3-6]. To ensure optimal obstetric management, it is required to have reliable information about gestational age,[7, 8] . Upon the following, one's knowledge of gestational age is again required for seeking proper care in right time including evaluation of fetal growth and management of preterm and post-term pregnancies,[7, 9]. Last menstrual Period (LMP) has been widely accepted for calculating the Expected Date of Delivery (EDD),[7, 10]. Therefore, recalling LMP is important from both obstetric and pediatric point of view which affects both mother and child,[10-13]. Assessment of accurate date and factors associated with LMP is related with prospect optimal prenatal care, screen for any chance of pre-eclampsia, fetal growth restriction (FGR) and prematurity,[10-14]. Approximately, 85% global preterm births occur in Asia and Africa,[15, 16]. Therefore, for best possible pregnancy outcomes for mothers and babies, it is imperative to detect pregnancy as early as possible since within the first eight weeks, the development of embryonic organ systems concludes and in this period the conceptus is more susceptible to serious malformations,[17].

For low and middle-income countries, assessing the gestational age by recalling the LMP is a simple and common method. In low-resource settings such as Bangladesh where limited information or technical knowledge is routinely available, healthcare workers often determine gestational age of newborns by relying on LMP,[18]. World Health Organization (WHO) also recommends recalling LMP for detecting gestational age as it is a simple and low-cost method,[19, 20]. Though LMP is used to detect gestational age, but it comes with great chances of recall error such as misinforming the LMP date and it has also a tendency to overestimate the duration of pregnancy,[14, 20]. Studies found that 15-45% women are not able to recall their

For peer review only - http://bmjopen.bmj.com/site/about/guidelines.xhtml

LMP accurately,[14, 20-22] . Again, one study conducted in Washington DC, USA showed that about 56% women could accurately recall their LMP date,[22] . However, due to the shortage of data on the prevalence of LMP recall among Bangladeshi women, we could not explore the current national situation.

One of the widely used methods of recalling LMP is calendar method,[4] .To use calendar accurately, women should have appropriate calendar literacy. There is a dearth of information about calendar literacy and factors associated with it. Prevalence of LMP recall and its associated factors are also not well documented.

The purpose of this paper is to explore the factors associated with calendar literacy and LMP recall, how women track, and how calendar literacy contributes in tracking menstruation dates, rate of recalling LMP.

Materials and Methods

Study design and settings

This was a cross sectional study conducted in both rural and urban areas. We had two study sites in rural areas and one in urban area from three Northern districts of Bangladesh. Rural areas were Chirirbandar and Saidpur sub-districts from Dinajpur and Nilphamari respectively and urban area was Rajshahi City Corporation. We conducted this survey during August to November 2016. An expert team was involved in preparing survey tool and questionnaires were developed based on research question and objectives of the study. Study investigators trained all the data collectors on data collection tool along with the pre-testing procedure. After pre-testing, we incorporated the feedbacks into the final version of the tool. After training and orientation, Research Assistants conducted the data collection in their assigned areas. Interviewers visited every household according to the eligibility criteria. The survey was administered through face-to-face interviews with women. This study had no more than minimal risk to study participants. We obtained written consent from each of the participants prior to the interviews. We received approval from Institutional Review Board (IRB) before data collection in the field.

For peer review only - http://bmjopen.bmj.com/site/about/guidelines.xhtml

Sampling and study participants

We applied two stages cluster sampling to select study participants in the study area. We considered catchment areas of government and non-government Community Based Health Worker (CBHW) as applicable both in rural and urban sites maintaining similar population coverage. In the first stage, we randomly selected one sub-district from two rural districts and 10 wards (lowest administrative unit of city area) from Rajshahi City Corporation. Then, we randomly selected 6 clusters (CBHW's catchment area) out of 12 in Chirirbandar sub-district, 6 clusters out of 12 clusters in Saidpur sub-district and 6 clusters from 10 clusters in Rajshahi. In each study area, we were supposed to conduct interviews with 900 women and finally, we completed 2,731 interviews from the three study areas. We enrolled women who had delivery outcomes of live birth in last one year prior to conducting interviews. Following were the inclusion criteria for the study enrolment: (1) had a live birth outcome in last 1 year, (2) passed 28 or more days after last delivery, (3) could hear, see and speak (4) had permanent residence (5) gave permission to interview within two days.

Measures

All the information given by the mothers were self-reported. The primary outcome variables for this study were calendar literacy and LMP recall. Calendar literacy was assessed using two screening questions and was categorized as literate or illiterate. The followings were the questions: 1.could mention the current date correctly according to any calendar (Bengali/English/Hijri calendar) - i) yes or ii) no 2. Could mark the current date correctly according to any calendar - i) yes or ii) no. 3 each question was scored by participants on a scale of 1 (for yes) or 0 (for no). We also calculated for those who marked current day, month, year and date with respective Bengali, English and Hijri calendar by themselves. To measure the rates of recalling LMP, we considered 1st Day of Last Menstrual Period. The women were asked about LMP date of their menstrual cycle they had last time prior to interview date. Study also includes socio-demographic characteristics such as age, place of residence, educational status, employment and economic status. Regarding age and education of the respondents, we considered completed years of age and passing years of schooling. By gravida we meant the total number confirmed pregnancy that our participants had. We also collected the history of their pregnancies. For measuring the economic status the participants were also asked about their

For peer review only - http://bmjopen.bmj.com/site/about/guidelines.xhtml

household assets, if they owned any of these assets and the positive responses was summed to derive the assets variable which was categorized from 1 to 5 indexes- poorer, poor, middle, rich and richest.

Statistical analysis

Quantitative analysis was performed using the statistical software package STATA, version 13. Descriptive analyses were conducted to describe characteristics of the study participants and also for calendar literacy and LMP recall. Mean and frequencies were computed for continuous and categorical variables, respectively, considering 95% confidence interval. Principal Component Analysis method was applied to conduct wealth index analysis. Logistic regression analysis was performed to examine the association between calendar literacy and different covariates. Similarly to see the effect of covariates on LMP recall, we executed logistic regression. Multinomial logistic regression was used to examine the association between calendar literacy and LMP recall. Both regression analyses were controlled for socio-demographic characteristics including age, place of residence, education level, employment status and economic status since these factors might influence the calendar literacy and recall of LMP. All statistically significant results were reported at p value <0.05.

Results

Socio-demographic characteristics of the study participants

Table 1 below shows the socio-demographic characteristics of the study participants. During the survey, most of the participants (62%) belonged to the age group of 20 to 29 years followed by 22%, who were below 20 years of age. Overall 80% women had education of more than 5 years and 51% had passed junior secondary level (class 8 and above). Among the participants, 95% of women were homemakers. Fifty percent of the respondents mentioned that they had experienced two pregnancies (median 2).

For peer review only - http://bmjopen.bmj.com/site/about/guidelines.xhtml

Table 1 Socio demographic characteristics of the survey participants

Traits	Total n =2731 (%)
**Age in years**	
≤19	609(22.3)
20-29	1693(62.0)
≥30	429(15.7)
**Participant years in schooling**	
<5	541 (19.8)
5-7	796 (29.2)
≥ 8	1394 (51.0)
**Residence**	
Urban	915 (33.5)
Rural	1816 (66.5)
**Primary occupation of the participant**	
Homemaker	2583(94.6)
Employed*	148(5.4)
**Gravida**	
1	1038 (38.0)
≥2	1693 (62.0)
**Median no. of pregnancies (Gravida)**	2
**Wealth Index**	
Poorer	547(20.0)
Poor	546(20.0)
Middle	546(20.0)
Rich	546(20.0)
Richest	546(20.0)

For peer review only

For peer review only - http://bmjopen.bmj.com/site/about/guidelines.xhtml

Calendar literacy

The participants were asked whether they could tell name of the present day and month, year and the date (the current day of interview) according to Bengali and English calendar. From the findings related to calendar information and literacy in "Fig 1 Calendar literacy" almost all the participants (97%) were able to tell the Bengali name of the days of week instantly which is almost same in both rural and urban areas. On the other hand, 45% participants were able to tell English name of the days of the week while participants from urban areas could mention better than rural areas (56% Vs 40%). Majority of the urban women were able to name the English calendar months (77%) rather than the Bengali calendar months (44%). More rural women (64%) mentioned Bengali calendar months compared to English calendar months (54%). However, the name of years was mentioned accurately based on the English calendar (urban 85% Vs rural 72%) by majority of the participants whereas only few participants could name those according to the Bengali calendar (urban 7% Vs rural 6%). In case of remembering date, majority participants both in urban and rural areas (78% and 58%) were able to correctly mention the present date based on the English calendar while 12% of total participants told the date using the Bengali calendar.

Table 2 below shows that, most of the women (63%) were capable of using any type of calendar. The participants who used calendars for calculating dates, among them overall 61% women were able to use English calendars correctly whereas only 4% of them were able to use Hijri calendar (Arabic calendar) and about 16% of them were able to use Bengali calendar correctly. More than half of the respondents (58.4%) had calendar at their home. According to purpose of using calendar, about 44% of women reported to use it for calculating days, dates and followed by 10% who used it to track the dates of menstrual period.

For peer review only - http://bmjopen.bmj.com/site/about/guidelines.xhtml

Table 2 Information regarding calendar literacy

Traits	Total n=2731(%)
Could mark date from Bengali calendar	448(16.4)
Could mark date from English calendar	1668(61.1)
Could mark date from Hijri calendar	114(4.2)
Could mark date from any calendar	1730(63.4)
**Availability of calendar at home**	
Yes	1594(58.4)
No	1137(41.6)
**Purpose of using calendar***	
To calculate days and dates	1204(44.1)
To track the dates of menstrual period	268(9.8)
To remember the special day (Hat day Eid/ Puja /selling certain goods)	364(13.3)
To remember date of tuition fee of children¨	85(3.1)
To remember the Installment day	61(2.2)
Others*[1]	39(1.4)
* refers multiple responses	
*[1] refers child date of birth, calculate moonlight etc	

Supplementary Table 1 indicates participants from age group 20-29, most of them (67%) were calendar literate. The women who were involved in earning activities had more (81%) literacy regarding calendar. The women who had passed class 8 and above most of them (89%) were calendar literate. The women who did not have schooling up to primary (class 5), 90% of them were calendar illiterate. About 81% of employed women were literate regarding calendar.

Educational status of calendar literate women

In "Fig 2 Educational status of calendar literate women" we explored the relationship between calendar literacy and educational background of the study participants. As mentioned above in the Table 2, about 63% of women could mark any calendar correctly. The women who could mark the correct date in the calendar, most of them (72%) had passed class 8 or above. Only a few (3%) of them had no education or passed up to class 4.

For peer review only - http://bmjopen.bmj.com/site/about/guidelines.xhtml

Calendar literacy and its associated covariates

Table 3 below presents that almost all the indicators were crudely associated with higher prevalence of literate woman regarding calendar. After adjustment, the odds ratio of calendar literate women who passed class 8 and above was 34.5 times (adj. OR 34.5, 95% CI: 24.00, 49.57) higher than women who did not pass primary. The women who belong to the top two wealth quintiles (i.e. rich adj. OR 2.75, 95% CI: 1.83, 4.11 and richest adj. OR 3.15, 95% CI: 1.88, 5.29) were 2.75 and 3.15 times respectively highly calendar literate than poor women.

For peer review

Table 3 Crude and adjusted OR for the factors associated with calendar literacy

Covariates	cOR 95% CI	aOR	P value	95% CI
**Constant**		0.05	<0.001*	.03 to .08
**Age group of women**				
≤19 years	1.2 0.96 to 1.58	0.95	0.805	.61 to 1.47
20 to 29 years	1.7 1.4 to 2.1	1.20	0.313	.84 to 1.69
≥ 30 years	1.0	1.0		
**Years of schooling**				
<5	1.0	1.0		
5-7	11.4 8.3 to 15.7	8.35	<0.001*	5.94 to 11.73
≥8	76.3 54.8 to 106.3	34.46	<0.001*	23.96 to 49.57
**Residence**				
Rural	1.0	1.0		
Urban	2.5 2.1 to 3.0	1.20	0.301	.85 to 1.69
**Primary occupation of the participant**				
Homemaker	1.0	1.0		
Employed*	2.6 1.7 to 3.9	1.83	0.052	.99 to 3.38
**Gravida**				
1	1.9 1.6 to 2.3	1.21	0.183	.91 to 1.60
≥2	1.0	1.0		

For peer review only - http://bmjopen.bmj.com/site/about/guidelines.xhtml

Covariates	cOR 95% CI	aOR	P value	95% CI
**Constant**		0.05	<0.001*	.03 to .08
**Wealth Index**				
Poorer	1.0	1.0		
Poor	2.2 1.7 to 2.8	1.71	0.001*	1.25 to 2.34
Middle	2.9 2.3 to 3.7	1.28	0.139	.92 to 1.77
Rich	6.4 4.9 to 8.3	2.75	<0.001*	1.83 to 4.11
Richest	15.3 11.1 to 21.1	3.15	<0.001*	1.88 to 5.29
**Availability of calendar at home**				
No	1.0	1.0		
Yes	4.8 4.0 to 5.6	0.65	0.005*	.48 to .88
**Purpose of use calendar**				
To calculate days and dates/ installation day	12.5 10.1 to 15.5	6.41	<0.001*	4.57 to 8.98
To track the dates of menstrual period	5.8 3.9 to 8.8	1.08	0.761	.66 to 1.76

Significant p value ≤0.05.
aOR, adjusted OR; cOR, crude OR.
Model adjusted for all indicators in the table 3
CI – Confidence interval
*refers Service/Business/Handicraft/Agriculture/Farm/fishing, and day labor, etc.

LMP recall

The recalling status of LMP in the context of last pregnancy and recent LMP varied a little bit in both rural and urban areas. Supplementary Table 2 presents that a fragment more than half (53%) of the participants could recall their recent LMP dates.

Most of the participants (59%) who were likely to recall their recent LMP date belonged to the age group 13- 19 years. There was a statistical significant ($p<0.01$) association between

For peer review only - http://bmjopen.bmj.com/site/about/guidelines.xhtml

education and the respondents' recalling status. The respondents who had passed class 8 and above, most of them (60%) could recall the LMP date. The women who were calendar literate, about 60% of them could recall their recent LMPs and there was a significant (p-value<0.01) association between calendar literacy and LMP recall.

LMP recall and its associated covariates

Table 4 below presents that almost all the indicators were crudely associated with higher prevalence of recalling recent LMP. After adjustment, the odds ratio of recalling recent LMP for women who passed class 8 or above was 1.39 times (adj. OR 1.39, 95% CI: 1.05, 1.84) higher than women who did not pass primary. Similarly, the richest women were more likely to recall recent LMP than poor women (rich: adj. OR 1.51, 95% CI: 1.12, 2.03 and richest: 1.61, 95% CI: 1.13, 2.30). The odds ratio of recalling LMP for calendar literate women was 1.59 times (adj. OR 1.59, 95% CI: 1.28, 1.98) higher than calendar illiterate women.

Table 4 Crude and adjusted OR for the factors associated with LMP recall

Covariates	cOR 95% CI	aOR	P value	95% CI
**Constant**		0.41	<0.001*	0.31 to 0.55
**Age group of women**				
≤19 years	1.84 (1.43 to 2.36)	1.11	0.517	0.82 to 1.5
20 to 29 years	1.45 (1.17 to 1.8)	1.10	0.408	0.88 to 1.38
≥ 30 years	1.0	1.0		
**Years of schooling**				
<5	1.0	1.0		
5-7	1.76 (1.41 to 2.2)	1.26	0.072	0.98 to 1.61
≥8	2.56 (2.08 to 3.14)	1.39	0.022*	1.05 to 1.84
**Residence**				
Rural	1.0	1.0		
Urban	1.09 (0.93 to 1.28)	0.80	0.054	0.63 to 1
**Primary occupation of the participant**				
Homemaker	1.0	1.0		
Employed*	1.08 (0.77 to 1.5)	0.85	0.37	0.6 to 1.21

For peer review only - http://bmjopen.bmj.com/site/about/guidelines.xhtml

Covariates	cOR 95% CI	aOR	P value	95% CI
**Constant**		0.41	<0.001*	0.31 to 0.55
**Gravida**				
1	2.14 (1.82 to 2.51)	1.88	<0.001*	1.55 to 2.29
≥2	1.0	1.0		
**Wealth Index**				
Poorer	1.0	1.0		
Poor	1.48 (1.17 to 1.88)	1.28	0.05*	1 to 1.64
Middle	1.53 (1.2 to 1.94)	1.28	0.061	0.99 to 1.66
Rich	1.72 (1.35 to 2.18)	1.51	0.007*	1.12 to 2.03
Richest	1.92 (1.51 to 2.45)	1.61	0.008*	1.13 to 2.3
**Availability of calendar at home**				
No	1.0	1.0		
Yes	1.35 (1.15 to 1.57)	0.95	0.658	0.74 to 1.21
**Purpose of use calendar**				
**To calculate days and dates/ installation day**				
No	1.0	1.0		
Yes	1.48 (1.27 to 1.72)	0.94	0.668	0.73 to 1.23
**To track the dates of menstrual period**				
No	1.0	1.0		
Yes	1.56 (1.2 to 2.02)	1.23	0.154	0.93 to 1.63
**Calendar literacy**				
Literate	2.12 (1.81 to 2.49)	1.59	<0.001*	1.28 to 1.98
Illiterate	1.0	1.0		

Significant p value ≤0.05
aOR, adjusted OR; cOR, crude OR.
Model adjusted for all indicators in the table 4
CI – Confidence interval
*refers Service/Business/Handicraft/Agriculture/Farm/fishing, and day labor, etc.

For peer review only - http://bmjopen.bmj.com/site/about/guidelines.xhtml

Discussion

This study determined how calendar literacy is associated with LMP recall. We found that, the calendar literacy rate was much higher in urban areas than that of in rural areas. Most of the participants could tell the name of years in English over Bengali. However, names of the weekly days were more commonly answered in Bengali. Approximately, more than half of the women were able to read or mark a calendar correctly. Furthermore, among the women who were using calendar for menstrual tracking, most of them figured as calendar literate. Our study also found significant relation between LMP recall and education level. Women with schooling of 8 years or above were more able to recall their LMP than the women with primary education. We further found that women who were calendar literate can recall LMP 1.59 times higher than those who were calendar illiterate. We also found association between LMP recall and social status, where rich women were more likely to recall LMP more accurately than poor women.

To our best knowledge, ours one is the first study reporting association of calendar literacy with LMP recall in Bangladesh, conducted both in urban and rural settings, although the validity of our findings are yet to be compared with others' findings since there has not yet been any other study published among the population nor we found any systematic review or meta-analysis on Bangladesh reporting the pooled prevalence of either calendar literacy or LMP recall.

A study about using home calendar was done in Bangladesh in 2016. The study examined the significance of LMP to measure the gestational age by using home calendar and explored how it differed from ultrasound result. That study reported to validate the use of prospectively collected LMP for estimating gestational age in a low-income, rural setting in Bangladesh,[23].

Another study conducted in 2005, compared the recalling dates of LMPs with prospective recorded dates. The study found that most women were able to recall their LMP accurately. But once the length of recall went beyond three weeks, women generally overestimated the dates,[22].

A study conducted in Bangladesh presented that, LMP recalling is a valid and highly-feasible estimate of gestational age where ultrasound is not routinely available,[18, 24]. Estimating

For peer review only - http://bmjopen.bmj.com/site/about/guidelines.xhtml

gestational age from LMP requires good maternal recall of dates and cycle characteristics, which may be a function of literacy,[24]. A multi-country trial showed 99.9% women could provide an exact date of their LMP and among that 92.4% of LMP dates were within a week,[25]. LMP can predict gestational age well if cycle characteristics and the date of onset of the last menstrual bleed can be clearly established, yet this has been proven difficult in many LMIC settings,[26]. Another study conducted in rural Bangladesh also conveyed that in some settings, LMP dates are greatly considered to estimate gestational age accurately,[16]. Two other studies also presented evidence that it is a reliable way to calculate gestational age where one study had precision level of 86-90% for LMP's accuracy and reliability,[18, 27]. Despite the limitations, studies conducted in different geographical setting such as Bangladesh and Guatemala have recommended LMP to be the chosen method for determining gestational age in low-resource settings,[20, 28, 29].

Many scholarly articles found that calendar literacy has ability to trigger tracking LMP date efficiently which is essential because it helps to calculate gestational age,[24, 30-32]. In many studies related to preterm birth, fetal growth restriction and maternal health we found dependency and consideration of LMP date and prioritizing it to determine gestational age,[24, 30-32]. In Bangladesh, tracking one's menstrual cycle using a calendar is a concept which is yet to become common and practiced. If women are calendar literate, they will be able to have a clear idea about their menstrual cycle, monitor their cycle and detect any menstrual abnormalities. In addition to these, they will be able to calculate the gestational age properly and identify pregnancy at an earlier stage. Early identification of pregnancy can lead to proper and timely utilization of ANC. This would prevent complications during pregnancy and delivery for the mothers and the babies thus increase utilization of PNC to improve birth outcomes.

Another study affirms that woman who received adequate number of ANC at the right times by a skilled attendant was most likely to have the delivery by a skilled attendant which contributes significantly to improve maternal and child health and smoothen the path to achieve SDG goals,[33].

For peer review only - http://bmjopen.bmj.com/site/about/guidelines.xhtml

This study was conducted in the northern part of Bangladesh considering sample survey estimates. As there are regional differences in terms of socio-demographic and health outcome indicators, this study finding cannot be generalized for the nationwide estimates.

Conclusion and recommendation

Recalling LMP accurately is very crucial for maternal health as it helps to calculate the gestational age accurately. It also helps to early detection which can have influence on early initiation of pregnancy care. We found significant relation between calendar literacy and LMP recall. Though the calendar literacy rate is good, however; practice of LMP tracking using calendar is still low. Besides calendar literacy, factors like education, place of residence and socio-economic status have a great influence on LMP recall too. Mounting all the relevant evidence, we summed up; calendar literacy is an opportunity to introduce a simple tool or calendar through existing health system to help women with a tool to track their LMPs regularly to improve maternal health outcomes in Bangladesh.

Funding statement

This research protocol was funded by Bill & Melinda Gates Foundation. The awarded BMGF grant number is OPP1146943.

Competing interests

The authors declare that they have no competing interests.

Patient and Public Involvement

Patients or the public were not involved in the design, or conduct, or reporting, or dissemination plans of this study

Authors' contributions

BKS, MR, DKM, MKM, AA and AR conceived and designed the experiments. BKS, MR, TR2, JJK, MH and MSM performed the experiments. BKS, TR1, TR2, FA, MH, SNM, AA and AR

For peer review only - http://bmjopen.bmj.com/site/about/guidelines.xhtml

were involved in analyzing data. BKS, MR, TR2, JJK, TR1, MH, FA, MSM and FR contributed to developing the tools. BKS, MR, TR1, TR2, FR, SNM, DKM, MKM, AA and AR wrote the paper.

Acknowledgement

icddr,b acknowledges with gratitude the commitment of Bill & Melinda Gates Foundation to its research efforts. We are grateful to our study participants for their spontaneous participation and sincere commitment to fulfill the research endeavor. icddr,b is also grateful to the Governments of Bangladesh, Canada, Sweden and the UK for providing core/ unrestricted support.

References

1 Alkema L, Doris C, Hogan D, et al., *Global, regional, and national levels and trends in maternal mortality between 1990 and 2015, with scenario-based projections to 2030: a systematic analysis by the UN Maternal Mortality Estimation Inter-Agency Group.* The Lancet, 2016. **387**(10017): p. 462-474.

2 Wanjira C, Mwangi M, Mathenge E, et al., *Delivery practices and associated factors among mothers seeking child welfare services in selected health facilities in Nyandarua South District, Kenya.* BMC public health, 2011. **11**(1): p. 360.

3 Dunson D.B, Colombo B, and Baird D.D, *Changes with age in the level and duration of fertility in the menstrual cycle.* Human reproduction, 2002. **17**(5): p. 1399-1403.

4 Sohda S, Suzuki K, and Igari I, *Relationship between the menstrual cycle and timing of ovulation revealed by new protocols: analysis of data from a self-tracking health app.* Journal of medical Internet research, 2017. **19**(11): p. e391.

5 Stanford J.B, White Jr G.L., and Hatasaka H, *Timing intercourse to achieve pregnancy: current evidence.* Obstetrics & Gynecology, 2002. **100**(6): p. 1333-1341.

6 Wilcox, A.J, Weinberg C.R, and Baird D.D, *Timing of Sexual Intercourse in Relation to Ovulation: Effects on the Probability of Conception, Survival of the Pregnancy, and the Sex of the Baby.* Obstetrical & gynecological survey, 1996. **51**(6): p. 357-358.

7 Ghani A, Nahar A, Sultana N, et al., *Prediction of Gestational age by Last Menstrual Period (LMP) in Comparison to Ultrasonography (USG).* Journal of Shaheed Suhrawardy Medical College, 2014. **6**(2): p. 82-86.

For peer review only - http://bmjopen.bmj.com/site/about/guidelines.xhtml

8 Tunon K, Eik-Nes SH, and Grøttum P, *A comparison between ultrasound and a reliable last menstrual period as predictors of the day of delivery in 15 000 examinations.* Ultrasound in Obstetrics and Gynecology: The Official Journal of the International Society of Ultrasound in Obstetrics and Gynecology, 1996. **8**(3): p. 178-185.

9 Olesen A.W, and Thomsen S.G, *Prediction of delivery date by sonography in the first and second trimesters.* Ultrasound in Obstetrics and Gynecology: The Official Journal of the International Society of Ultrasound in Obstetrics and Gynecology, 2006. **28**(3): p. 292-297.

10 Macaulay S, Buchmann E.J, Dunger D.B, et al., *Reliability and validity of last menstrual period for gestational age estimation in a low‐to‐middle‐income setting.* Journal of Obstetrics and Gynaecology Research, 2019. **45**(1): p. 217-225.

11 Kamana KC, Shakya S, and Zhang H, *Gestational diabetes mellitus and macrosomia: a literature review.* Annals of Nutrition and Metabolism, 2015. **66**(Suppl. 2): p. 14-20.

12 Cnattingius S, Villamor E, Lagerros YT, et al., *High birth weight and obesity—a vicious circle across generations.* International journal of obesity, 2012. **36**(10): p. 1320.

13 Salahuddin M, Pe'rez A, Ranjit N, et al., *Predictors of Severe Obesity in Low-Income, Predominantly Hispanic/Latino Children: The Texas Childhood Obesity Research Demonstration Study.* Preventing chronic disease, 2017. **14**: p. E141-E141.

14 van Oppenraaij RHF, Eilers PHC, Willemsen SP, et al., *Determinants of number‐specific recall error of last menstrual period: a retrospective cohort study.* BJOG: An International Journal of Obstetrics & Gynaecology, 2015. **122**(6): p. 835-841.

15 Beck S, Wojdyla D, Say L, et al., *The worldwide incidence of preterm birth: a systematic review of maternal mortality and morbidity.* Bulletin of the World Health Organization, 2010. **88**: p. 31-38.

16 Shah R, Mullany L.C, Darmstadt G.L, et al., *Incidence and risk factors of preterm birth in a rural Bangladeshi cohort.* BMC pediatrics, 2014. **14**(1): p. 112.

17 Poma P.A, *Early detection of pregnancy.* Journal of the National Medical Association, 1984. **76**(3): p. 305.

18 Rosenberg R.E, Ahmed ASM.N.U, Ahmed S, et al., *Determining gestational age in a low-resource setting: validity of last menstrual period.* Journal of health, population, and nutrition, 2009. **27**(3): p. 332.

For peer review only - http://bmjopen.bmj.com/site/about/guidelines.xhtml

19 *Home-based maternal records: guidelines for development, adaptation and evaluation.* 1994: World Health Organization.

20 Salam S.S, Ali N.B, Rahman A.E, et al., *Study protocol of a 4-parallel arm, superiority, community based cluster randomized controlled trial comparing paper and e-platform based interventions to improve accuracy of recall of last menstrual period (LMP) dates in rural Bangladesh.* BMC public health, 2018. **18**(1): p. 1359.

21 Waller D.K, Spears WD, Gu Y, et al., *Assessing number-specific error in the recall of onset of last menstrual period.* Paediatric and Perinatal Epidemiology, 2000. **14**(3): p. 263-267.

22 Wegienka G, and Baird D.D, *A comparison of recalled date of last menstrual period with prospectively recorded dates.* Journal of Women's Health, 2005. **14**(3): p. 248-252.

23 Gernand A.D, Paul R.R, Ullah B, et al., *A home calendar and recall method of last menstrual period for estimating gestational age in rural Bangladesh: a validation study.* Journal of Health, Population and Nutrition, 2016. **35**(1): p. 34.

24 Karl S, Suen C.SN, Unger H.W, et al., *Preterm or not–an evaluation of estimates of gestational age in a cohort of women from rural Papua New Guinea.* PloS one, 2015. **10**(5): p. e0124286.

25 Winikoff B, Ellertson C, Elul B, et al., *Acceptability and feasibility of early pregnancy termination by mifepristone-misoprostol: results of a large multicenter trial in the United States.* Archives of Family Medicine, 1998. **7**(4): p. 360.

26 Unger H, Thriemer K, Ley B, et al., *The assessment of gestational age: a comparison of different methods from a malaria pregnancy cohort in sub-Saharan Africa.* BMC pregnancy and childbirth, 2019. **19**(1): p. 12.

27 Ellertson C, Elul B, Ambardekar S, et al., *Accuracy of assessment of pregnancy duration by women seeking early abortions.* The Lancet, 2000. **355**(9207): p. 877-881.

28 Persson L.Å, Arifeen S, Ekstrom E, et al., *Effects of Prenatal Micronutrient and Early Food Supplementation on Maternal Hemoglobin, Birth Weight, and Infant Mortality Among Children in Bangladesh: The MINIMat Randomized Trial.* JAMA, 2012. **307**(19): p. 2050-2059.

29 Neufeld L.M, Haas J.D, Grajeda R, et al., *Last menstrual period provides the best estimate of gestation length for women in rural Guatemala.* Paediatric and perinatal epidemiology, 2006. **20**(4): p. 290-298.

For peer review only - http://bmjopen.bmj.com/site/about/guidelines.xhtml

30 Rashid H, Ma E, Ferdous F, et al., *First-trimester fetal growth restriction and the occurrence of miscarriage in rural Bangladesh: A prospective cohort study.* PloS one, 2017. **12**(7): p. e0181967.

31 Baqui A, Rosen HE, Lee A.CC, et al., *Preterm birth and neonatal mortality in a rural Bangladeshi cohort: implications for health programs.* Journal of Perinatology, 2013. **33**(12): p. 977.

32 Lee A.CC, Mullany L.C, Ladhani K, et al., *Validity of newborn clinical assessment to determine gestational age in Bangladesh.* Pediatrics, 2016. **138**(1): p. e20153303.

33 Al Kibria G.M, Ghosh S, Hossen S, et al., *Factors affecting deliveries attended by skilled birth attendants in Bangladesh.* Maternal health, neonatology and perinatology, 2017. **3**(1): p. 7.

For peer review only

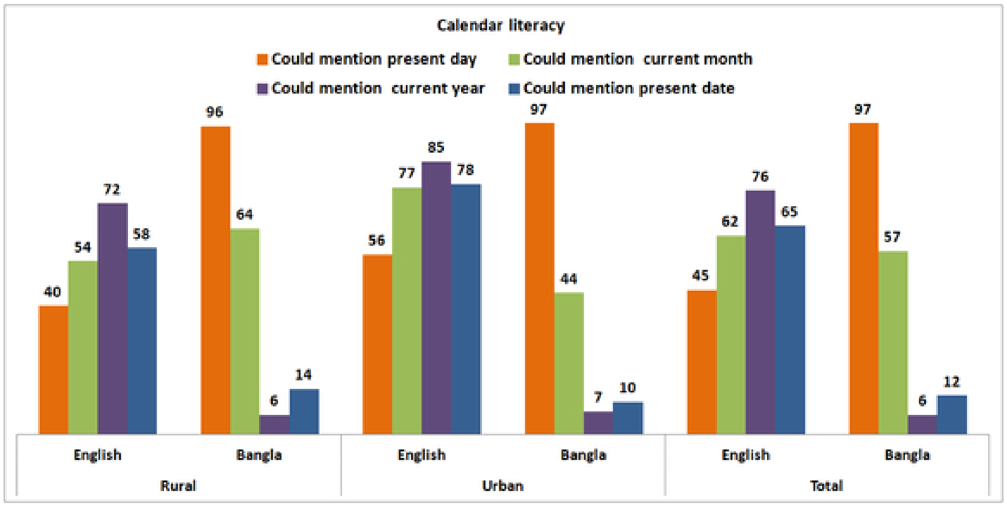

Fig 1 Calendar literacy

49x24mm (300 x 300 DPI)

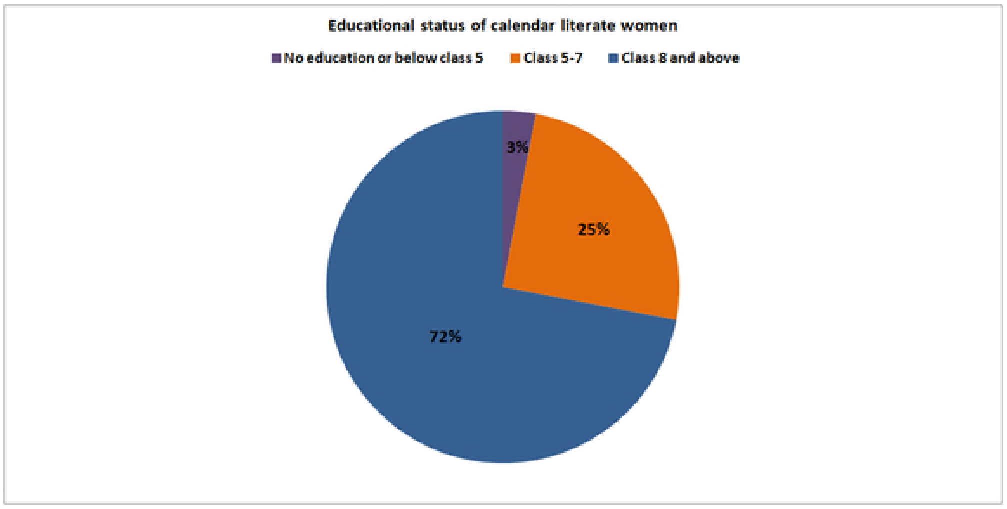

Fig 2 Educational status of calendar literate women

48x24mm (300 x 300 DPI)

Supplementary Table 1 Association between calendar literacy and demographic characteristics of the study participants

Traits	Calendar literacy		P-value
	Literate 1730 (%)	Illiterate 1001 (%)	
**Age in years**			
≤19	362(59.4)	247 (40.6)	
20-29	1135(67.0)	558 (33.0)	<0.001
≥30	233(54.3)	196 (45.7)	
**Years of schooling**			
<5	52 (9.6)	489 (90.4)	
5-7	437 (54.9)	359 (45.1)	<0.001
≥ 8	1241 (89.0)	153 (11.0)	
**Residence**			
Rural	1,029 (56.7)	787 (43.3)	<0.001
Urban	701 (76.6)	214 (23.4)	
**Primary occupation of the participant**			
Homemaker	1610 (62.3)	973 (37.7)	<0.001
Employed*	120 (81.1)	28 (18.9)	
**Gravida**			
1	751 (72.4)	287 (27.7)	<0.001
≥2	979 (57.8)	714 (42.2)	
**Wealth Index**			
Poorer	355(64.0)	192(35.1)	
Poor	251(46.0)	295(54.0)	
Middle	213(39.0)	333(61.0)	<0.001
Rich	123(22.5)	423(77.5)	
Richest	59(10.8)	487(89.2)	
**Availability of calendar at home**			
Yes	1244(78.0)	350(22.0)	<0.001
No	486(42.7)	651(57.3)	

Traits	Calendar literacy		P-value
	Literate 1730 (%)	Illiterate 1001 (%)	
**Purpose of use calendar***			
To calculate days and dates	1085(90.1)	119(9.9)	
To track the dates of menstrual period	241(89.9)	27(10.1)	
To remember the special day (Hat day Eid/ Puja /selling certain goods /)	342(94.0)	22(6.0)	<0.001
To remember date of tuition fee of children¨	77(90.6)	8(9.4)	
To remember the installment day	57(93.4)	4(6.6)	
Others*	36(92.3)	3(7.7)	
* refers multiple response			

Supplementary Table 2 Association between LMP recall and demographic characteristics

Traits	Recall recent LMP		P-value
	Yes	No	
**Age in years**			
≤19	359(58.9%)	250(41.1%)	
20-29	899(53.1%)	794(46.9%)	<0.001
30+	188(43.8%)	241(56.2%)	
**Years in schooling**			
<5	201(37.2%)	340(62.8%)	
5-7	406(51.0%)	390(49.0%)	<0.001
≥ 8	839(60.2%)	555(39.8%)	
**Residence**			
Rural	948(52.2%)	868(47.8%)	<0.001
Urban	498(54.4%)	417(45.6%)	
**Primary occupation of the participant**			
Housewife	1365(52.8%)	1218(47.2%)	<0.001
Others*	81(54.7%)	67(45.3%)	

Traits	Recall recent LMP		P-value
	Yes	No	
**Gravida**			
1	669(64.5%)	369(35.5%)	<0.001
≥2	777(45.9%)	916(54.1%)	
**Wealth Index**			
Poorer	235(43.0%)	312(57.0%)	<0.001
Poor	288(52.7%)	258(47.3%)	
Middle	292(53.5%)	254(46.5%)	
Rich	308(56.4%)	238(43.6%)	
Richest	323(59.2%)	223(40.8%)	
**Availability of calendar at home**			
Yes	893(56.0%)	701(44.0%)	<0.001
No	553(48.6%)	584(51.4%)	
**Purpose of use calendar***			
To calculate days and dates	703(58.4%)	501(41.6%)	0.042
To track the dates of menstrual period	168(62.7%)	100(37.3%)	
To remember the special day (Hat day Eid/ Puja /selling certain goods /)	288(65.5%)	152(34.5%)	
To remember date of tuition fee of children¨	55(57.9%)	40(42.1%)	
To remember the Installment day	33(54.1%)	28(45.9%)	
Others*	22(56.4%)	17(43.6%)	
**Calendar literacy**			
Literate	1034(59.8%)	696(40.2%)	<0.001
Illiterate	412(41.2%)	589(58.8%)	
**Track LMP date**			
Written	1394(53.9%)	1194(46.1%)	<0.001
Unwritten	52(36.4%)	91(63.6%)	
**Total**	1446 (53.0%)	1285 (47.0%)	

* refers multiple response

Research Checklist

Section/Topic	Item #	Description	Reported on page #
**Title and abstract**	1	(*a*) Study design within abstract	2
		(*b*) Summary of what was done and what was found	2
**Strength and Limitation**	2	Four bullet points on strength and limitation of this Study	3
**Introduction**			
Background/rationale	3	Scientific background and rationale of this study	4-5
Objectives	4	Specific objectives of this study	5
**Materials and Methods**			
Study design and settings	5	Study design, settings of the study, data collection period	5
Sampling and study participants	6	Sampling strategy, sample size, inclusion criteria of study participants	6
Measures	7	Primary outcome variables, Operational definition of outcome measures and methods of measurement	6-7
Statistical analysis	8	Descriptive statistics, logistic regression, multinomial regression, Principal Component Analysis are done for the statistical analysis	7
**Results**			
Socio-demographic characteristics of the study participants	9	Age, educational status, place of residence, occupation and wealth index of the study participants are presented	7-8
Descriptive data	10	Characteristics of study participants (eg Age, educational status, place of residence, occupation and wealth index)	8
Calendar literacy	11	Prevalence of calendar literacy of the study participants is presented	9-10
Educational status of calendar literate women	12	Distribution of educational status among calendar literate women is presented	10
LMP recall	13	Prevalence of LMP recall of the study participants	
Main results	14	(*a*) Crude and adjusted OR for the factors associated with calendar literacy with the precision of 95% confidence interval. Covariates such as: age, educational status, place of residence, occupation and wealth index were adjusted	11-12
		(*b*) Crude and adjusted OR for the factors associated with LMP recall with the precision of 95% confidence interval. Covariates such as: age, educational status, place of residence, occupation and wealth index were adjusted	13-14
**Discussion**			
Key results	15	Summary of key results	15
Limitations	16	Limitations of the study is described	15
Interpretation	17	Overall interpretation of results considering objectives, limitations, multiplicity of analyses, results from similar studies, and other relevant evidence	15-16
Generalisability	18	Discussion about generalisability of the study results	17
**Other information**			
Funding	19	Give the source of funding and the role of the funders for the present study and, if applicable, for the original study on which the present article is based	17

For peer review only

BMJ Open

Factors associated with calendar literacy and Last Menstrual Period (LMP) recall: a prospective programmatic implication to maternal health in Bangladesh

Journal:	*BMJ Open*
Manuscript ID	bmjopen-2020-036994.R1
Article Type:	Original research
Date Submitted by the Author:	27-Jul-2020
Complete List of Authors:	Sarker, Bidhan Krishna; International Centre for Diarrhoeal Disease Research Bangladesh, Maternal and Child Health Division<br>Rahman, Musfikur; International Centre for Diarrhoeal Disease Research Bangladesh, Maternal and Child Health Divison<br>Rahman, Tanjina ; International Centre for Diarrhoeal Disease Research Bangladesh, Maternal and Child Health Division<br>Rahman, Tawhidur; International Centre for Diarrhoeal Disease Research Bangladesh, Maternal and Child Health Division<br>Rahman, Fariya; International Centre for Diarrhoeal Disease Research Bangladesh, Maternal and Child Health Division<br>Khalil, Jubaida; International Centre for Diarrhoeal Disease Research Bangladesh, Maternal and Child Health Division<br>Hasan, Mehedi; International Centre for Diarrhoeal Disease Research Bangladesh, Maternal and Child Health Division<br>Mahfuz, Sadia; University of Western Sydney<br>Ahmmed, Faisal; International Centre for Diarrhoeal Disease Research Bangladesh, Infectious Diseases Division<br>Miah, Muhammad Salim; Shahjalal University of Science and Technology, Department of Anthropology<br>Ahmed , Anisuddin; International Centre for Diarrhoeal Disease Research Bangladesh<br>Mitra, Dipak; North South University, Public Health<br>Mridha, Malay Kanti; BRAC University James P Grant School of Public Health<br>Rahman, Anisur ; International Centre for Diarrhoeal Disease Research, Maternal and Child Health Division
**Primary Subject Heading**:	Public health
Secondary Subject Heading:	Obstetrics and gynaecology, Health policy, Health services research, Sexual health
Keywords:	Health policy < HEALTH SERVICES ADMINISTRATION & MANAGEMENT, OBSTETRICS, GYNAECOLOGY, EPIDEMIOLOGY, STATISTICS & RESEARCH METHODS, PUBLIC HEALTH

I, the Submitting Author has the right to grant and does grant on behalf of all authors of the Work (as defined in the below author licence), an exclusive licence and/or a non-exclusive licence for contributions from authors who are: i) UK Crown employees; ii) where BMJ has agreed a CC-BY licence shall apply, and/or iii) in accordance with the terms applicable for US Federal Government officers or employees acting as part of their official duties; on a worldwide, perpetual, irrevocable, royalty-free basis to BMJ Publishing Group Ltd ("BMJ") its licensees and where the relevant Journal is co-owned by BMJ to the co-owners of the Journal, to publish the Work in this journal and any other BMJ products and to exploit all rights, as set out in our licence.

The Submitting Author accepts and understands that any supply made under these terms is made by BMJ to the Submitting Author unless you are acting as an employee on behalf of your employer or a postgraduate student of an affiliated institution which is paying any applicable article publishing charge ("APC") for Open Access articles. Where the Submitting Author wishes to make the Work available on an Open Access basis (and intends to pay the relevant APC), the terms of reuse of such Open Access shall be governed by a Creative Commons licence – details of these licences and which Creative Commons licence will apply to this Work are set out in our licence referred to above.

Other than as permitted in any relevant BMJ Author's Self Archiving Policies, I confirm this Work has not been accepted for publication elsewhere, is not being considered for publication elsewhere and does not duplicate material already published. I confirm all authors consent to publication of this Work and authorise the granting of this licence.

For peer review only

Full Title

Factors associated with calendar literacy and Last Menstrual Period (LMP) recall: a prospective programmatic implication to maternal health in Bangladesh

Short title

Associated factors related to calendar literacy and LMP recall

# Corresponding author

Bidhan Krishna Sarker[1], Assistant Scientist, Maternal and Child Health Division, icddr,b. 68, Shaheed Tajuddin Ahmed Sarani, Mohakhali, Dhaka-1212, GPO Box-1000, Dhaka, Bangladesh, E-mail: bidhan@icddrb.org; bidhanicddrb@gmail.com

Authors

Bidhan Krishna Sarker[1], Musfikur Rahman[1], Tanjina Rahman[1], Tawhidur Rahman[1], Fariya Rahman[1], Jubaida Jahan Khalil[1], Mehedi Hasan[1], Sadia Nishat Mahfuz[2], Faisal Ahmmed[3], Muhammad Salim Miah[4], Anisuddin Ahmed[1], Dipak Kumar Mitra[5], Malay Kanti Mridha[6], Anisur Rahman[1]

Authors and Affiliations

[1]Maternal and Child Health Division, icddr,b. 68, Shaheed Tajuddin Ahmed Sarani, Mohakhali, Dhaka-1212, GPO Box-1000, Dhaka, Bangladesh

[2]MS student, University of Western Sydney, Australia

[3]Infectious Diseases Division, icddr,b. 68, Shaheed Tajuddin Ahmed Sarani, Mohakhali, Dhaka-1212, GPO Box-1000, Dhaka, Bangladesh

[4]Lecturer, Department of Anthropology, Shahjalal University of Science & Technology, Sylhet, Bangladesh

[5] Professor and Chair, Department of Public Health, North South University, Dhaka, Bangladesh

[6]Professor and Director of Centre of Excellence for Non-Communicable Diseases and Nutrition, James P Grant school of Public Health, BRAC University, Dhaka, Bangladesh

For peer review only - http://bmjopen.bmj.com/site/about/guidelines.xhtml

Abstract

Objective: To explore the prevalence of calendar literacy and Last Menstrual Period (LMP) recall along the factors associated with.

Design: We conducted cross-sectional survey during August-November, 2016.

Setting: Two rural sub-districts and one urban area from three Northern districts of Bangladesh.

Participants: We enrolled women who had live birth in last one year. In each study area, around 900 women were interviewed and finally, we completed 2,731 interviews.

Primary and secondary outcome measures: The primary outcome was LMP recall and secondary outcome was calendar literacy.

Results: Majority participants (65%) correctly mentioned the present date from English calendar while 12% told from Bengali calendar. We provided women with three calendars: Bengali, English and Hijri to mark the current date. Sixty-three percent women found as calendar literate who marked any of the calendars. Despite Bengali being state language, women were more capable of using English than Bengali Calendar (61% vs 16%). Among the calendar literate, 72% completed 8 or more years of schooling. Majority women (77%) in urban area were calendar literate while 57% in rural areas. Overall 53% women recalled recent LMP. Among the calendar literate, 60% recalled recent LMPs. Overall 58% households owned any of the calendars and only 10% used calendar to track LMP. Factors found significant with recalling recent LMP were: women completed 8 or more years of schooling (adj. OR 1.39), single gravida (adj. OR 1.88), richest wealth quintile (adj. OR 1.61) and calendar literacy (adj. OR 1.59).

Conclusions: Calendar use for tracking LMP found very low despite decent calendar literacy and availability. Calendar literacy found as one of the key factors associated with LMP recall. MNCH programmes should introduce and promote a simple tool like calendar at communities where ultrasound not available to ensure accurate LMP recall for early pregnancy registration and timely ANC coverage.

For peer review only - http://bmjopen.bmj.com/site/about/guidelines.xhtml

Strength and Limitation of this Study

1. This is the 1st large-scale study of its kind in Bangladesh reporting the prevalence of last menstrual period (LMP) recall with both urban and rural settings.
2. The study relied on the self-reported data but we also checked relevant documents (such as calendar) during our data collection to minimize the errors and we also cross-checked the validity of the data through re-interview.
3. We took into account of both government and non-government health service catchment areas maintaining similar population coverage and statistical rigor.
4. Since this study was conducted in the Northern part of Bangladesh where ANC coverage is higher, literacy rate is moderate and human resource gaps are low; hence the findings are subject to compare contextually.
5. There may be some other confounding factors (e.g. lack of awareness about importance of gestational age, irregular menstrual cycle, etc.) that were not explored in this study.

For peer review only

Introduction

Every day about 830 women die from pregnancy or childbirth-related complications around the world. Most of these deaths occur in low-resource settings and many of them are preventable.[1] The south Asia region alone accounts for approximately one-third of the global maternal and child deaths annually.[2] Globally, new target set by Sustainable Development Goals (SDG) for maternal health is to reduce mortality ratio less than 70 per 100,000 live births by 2030.[1] According to the recent national data, maternal mortality ratio in Bangladesh remains unchanged for the last decade (196 deaths per 100,000 live births-BMMS 2016) and a huge effort is needed to achieve the SDG targets.[3 4]

Pregnancy is an important part of women's life and women should have awareness regarding pregnancy care.[5-8] To ensure optimal pregnancy related care, it is required to have reliable information about gestational age.[9 10] To detect the gestational age, recall of Last Menstrual Period (LMP) plays an important role. Recalling of LMP is also known as "calendar method" of predicting the next ovulation date which was developed based on the recognition of cycles in the menstrual period and fertility, in which women record their menstrual cycles .[6] World Health Organization (WHO) also recommends recalling LMP for detecting gestational age because it is a simple and low-cost method.[11 12] LMP has been widely accepted for calculating the Expected Date of Delivery (EDD).[10 13] Studies suggest to use LMP recall method where ultrasound facilities are not readily available.[14 15] For low and middle-income countries, assessing the age of pregnancy by recalling the LMP is a simple and common method. For example, low-resource settings such as Bangladesh where healthcare workers often determine gestational age by relying on LMP.[14]

Though LMP is used to detect gestational age, but it has recall bias such as misinforming the LMP date or overestimating the duration of pregnancy.[12 16] Studies done in both developed and developing countries found that 15%-45% women are not able to recall their LMP accurately.[12 16-18] Study suggests that, calculation of LMP based on gestational age requires better maternal recall of dates and cycle characteristics which is somehow related to literacy.[15]

Accurate LMP recall helps to screen for any chance of pre-eclampsia, fetal growth restriction (FGR) and premature birth outcome.[13 16 19-21] Accurate LMP recall also helps to detect pregnancy

For peer review only - http://bmjopen.bmj.com/site/about/guidelines.xhtml

sooner that facilitates early initiation of pregnancy care. Initiation of pregnancy care at the right time is very important because the first eight weeks of gestational age is very vulnerable for mothers and embryos.[22]

However, there is a dearth of information regarding the prevalence of LMP recall and its associated factors in Bangladesh. Calendar literacy can be an integral part of recalling LMP accurately. The purpose of this paper is to explore the prevalence of calendar literacy and LMP recall and factors associated with. Findings from this study will help to improve Maternal, Neonatal and Child Health (MNCH) programmes to achieve SDG targets in resource-poor settings.

Materials and Methods

Study design and settings

This was a cross sectional study conducted in both rural and urban areas and the data collection period was from August to November, 2016. We had two study sites in rural areas and one in urban area from three Northern districts of Bangladesh. Rural areas were Chirirbandar and Saidpur sub-districts from Dinajpur and Nilphamari districts respectively and urban area was Rajshahi City Corporation. According to the Population and Housing Census (2011), Chirirbandar sub-district was consisted of 142 villages under 12 unions. The average size of population of each union and village was 24,375 and 2,060 respectively. The total population of Chirirbandar was 292,500 of which 146,619 were males and 145,881 were females.[23] Saidpur sub-district was consisted of 1 municipality and 5 unions with 39 villages. The average size of population of each union and village was 27,471 and 3,522 respectively. The total population of Saidpur was 264,461 of which 133,737 were males and 130,724 were females.[24] On the other hand, Rajshahi City Corporation was consisted of 4 thanas with 30 wards. The total population of the Rajshahi City Corporation was 449,756 of which 232,974 were males and 216,782 were females.[25] According to census data, female literacy rate was 42% in Dinajpur and 39% for both Nilphamari and Rajshahi districts.[26] Health Care Utilization data suggested that at least one ANC coverage in Dinajpur, Nilphamari and Rajshahi was 75%, 96% and 80% respectively.[27] Another data showed that there was medium vacancy (21% to 30%) in terms of healthcare personnel in Dinajpur, Nilphamari and Rajshahi

For peer review only - http://bmjopen.bmj.com/site/about/guidelines.xhtml

districts.[28] We considered moderate literacy rate, high ANC coverage and moderate vacancy for human resources to select the study areas.

Sampling and study participants

We applied two stages cluster sampling to select study participants in the study area. We considered catchment areas of government and non-government Community Based Health Worker (CBHW) as applicable both in rural and urban sites maintaining similar population coverage considered as clusters. In the first stage, we randomly selected one sub-district from two districts each and 10 wards (lowest administrative unit of city area) from Rajshahi City Corporation. Then, we randomly selected 6 clusters (CBHW's catchment area) out of 12 in Chirirbandar sub-district, 6 clusters out of 12 clusters in Saidpur sub-district and 6 clusters from 10 clusters in Rajshahi . We used conventional statistical method to calculate the sample size. Based on selected process indicators and assuming the improvement rate we calculated the sample size for the survey. The sample size was estimated using the formula proposed by Hayes and Bennett.[29] Considering the proportion of taking any ANC by the 1st trimester $p_{0=}$0.41 and $p_{1=}$0.57 and K=0.15, we needed the highest number of 150 mothers in each cluster including 5% non-response rate. Considering the proportion of taking at least one ANC from medically trained provider $p_{0=}$0.49 and $p_{1=}$0.66 and K=0.15, we needed the highest number of 6 cluster per area to assess the change in the indicators that was least known at baseline. For each study area the sample size was calculated 900 and thus the total required sample size was 2700 (150×6×3).

We recruited study participants through EPI method. At first, the starting point was selected to start data collection in the selected clusters. We consulted with the community people to determine the midpoint of each cluster. While ensuring randomization to begin interviewing eligible participants, we used bottle spinning method. A bottle was spun at the midpoint of each cluster to pick a random direction to start searching study participants.[30 31] Afterwards, every household on next door basis was visited as per bottle indicated direction and interviewers identified eligible participants for interviewing. Data collection continued until the cluster's sample size was met. During household visit, if any eligible woman was absent then data collectors tried at least two more times to interview her. We completed 2,731 interviews from the three study areas. We enrolled women who had delivery outcomes of live birth in last one year prior to conducting

For peer review only - http://bmjopen.bmj.com/site/about/guidelines.xhtml

interviews. Followings were the inclusion criteria for the study participant's enrolment: (1) had a live birth outcome in last 1 year, (2) past 28 or more days after last delivery, (3) could hear, see and speak (4) had permanent residence in the study area (5) gave permission to interview within two days.

Data collection

Survey tool development

We used semi-structured questionnaire to collect data through face-to-face interviews with women. A team of experienced investigators from icddr,b were responsible to develop this survey tool. Based on the research objectives and the expected outcome variables of the study, research team considered the relevant indicators to design the tool. As part of designing the tool, investigators did the desk review of relevant literature such as- Bangladesh Demographic and Health Survey, Bangladesh Maternal Mortality Survey and other similar survey conducted by icddr,b, national and WHO guidelines, etc. After formulating the tool, a pre-test was done to check the design of the questionnaire how it works in practice. After addressing all the problems related to the survey tool, the data collection team started collecting data. In the survey tool, we included the basic individual and household characteristics of the study participants (age, religion, education, employment status, income, household asset, place of residence, etc.); reproductive and obstetric information of the participants (age at first marriage, gravida, para, living children, pregnancy history, LMP date, etc.), knowledge and practice of calendar literacy (able to mention correctly Bengali and English day, month, year and date which is related to their knowledge; able to mark Bengali/English/Hijri date in the calendar, etc. which is related to their practice).

Staff recruitment and training

A team of efficient and experienced Field Research Assistants (FRA) were involved in data collection. FRAs had minimum Bachelor's degree with at least two years' previous experience on relevant field were recruited as an interviewer for this study. Preference was given to individuals who worked in similar positions in large scale surveys. After recruitment, the interviewers were trained and standardized on the survey tool to collect data. During training sessions key persons from the research team were present to discuss objectives of the study so that the data collection team can easily avoid any confusion. A training manual was developed for the interviewers that

For peer review only - http://bmjopen.bmj.com/site/about/guidelines.xhtml

included interviewing techniques and specific instructions for each of the data collection questionnaire and checklist.

Data management and quality assurance

A project research physician (PRP) and a research investigator (RI) coordinated the data collection team and data quality was ensured by an efficient team led by experienced team leaders. The team leaders were responsible for ensuring quality data and completeness of the interview during data collection period on a regular basis. The team leaders were solely responsible to check the completeness of every interview on spot after the data collection which was supervised through day to day monitoring. Again, the accuracy and validity of data was checked through re-interview done by the team leaders, PRP and RI as per monitoring plan. We re-interviewed almost one-third of the total interviewees to ensure the quality and validity of the data. Expert programmers of Maternal and Child Health Division (MCHD) were recruited to design a database template for the data entry systematic. Programming team used Dot net (Version-10) software for data template design as appropriate. Data template was designed in such a way that while entering data none of the variable could go missing. To avoid the entry mistakes, skipping options were also maintained strictly and logically. An expert data management team was engaged for entering all the data through online database. The data management team simultaneously entered both pre coded and post coded data. For post-coding of data, the research team was closely involved with the data management team.

Ethics

icddr,b have rigorous processes to meet research integrity and ethics which is called Institutional Review Board (IRB). icddr,b's IRB consists of four mandatory committees involving internal and external topic experts: Research Review Committee (RRC), Ethical Review Committee (ERC), Animal Experimentation Ethics Committee (AEEC) and Programme Coordination Committee (PCC). icddr,b attaches great importance to ensuring that all research protocols conducted by its scientists are scientifically sound and meet international ethical standards. To achieve this, in addition to review at the Centre level and by at least two external reviewers, all research protocols are subject to review by at least two of three committees: RRC, ERC (protocols involving human subjects), and the AEEC (protocols involving animals).

For peer review only - http://bmjopen.bmj.com/site/about/guidelines.xhtml

RRC has the authority to approve, suggest modifications to, or disapprove of any proposed research protocol. Any change to an approved research protocol also needs approval of the RRC. No research protocol can be implemented or proceed for consideration by the ERC or the AEEC without the RRC's approval.

The ERC's approval is required for all protocols involving human subjects. It is an independent body committed to protecting the rights of the people who participate in research protocols conducted under the auspices of icddr,b. A subcommittee of the ERC, the Data Safety Monitoring Board (DSMB), periodically reviews and evaluates the accumulated study and makes recommendations to the ERC concerning the continuation, modification or termination of the study.

We acquired both RRC and ERC approval from Institutional Review Board (IRB) of icddr,b before we start collecting data. During data collection period, we acquired written informed consent from all the participants after ensuring that none of them are exposed to minimal risk while participating in this study. Since all our participants were already married by then, we did not need to take consent for the minors from their guardian or parents as per IRB requirements.

Variables and Measures

All the information given by the mothers were self-reported. The primary outcome variable was LMP recall and the secondary outcome was calendar literacy. The rest of the variables were considered as independent variables. The description of all variables is given below-

For peer review only - http://bmjopen.bmj.com/site/about/guidelines.xhtml

List of variables	Explanation
LMP recall (Primary outcome variable)	We considered the 1st Day of Last Menstrual Period. The women were asked about LMP date of their menstrual cycle that they had last time prior to interview date.
Calendar literacy (Secondary outcome variable)	We asked study participants to mark the current date for 3 different types of calendars (Bengali, Hijri and English). If study participants marked it correctly, we coded 1 for correct answer and 0 for wrong answer. We considered women as calendar literate, if she could correctly mark the current date using any of the provided calendars.
Mentioned current day, month, year and date	Women were asked whether they could tell name of the present day, month, year and the date (the current day of interview)
Age in years	Considered completed years of age and categorized into complete years of aged ≤19, 20-29, ≥30
Years of schooling	Considered completed years of schooling and transformed it into three categories: 1. <5 who did not complete 5 years of schooling, 2. 5-7 who completed 5 to 7 years of schooling, 3. ≥8 who completed 8 or more years of schooling.
Place of residence	Considered permanent residence where the women were living permanently. We had rural and urban areas as place of residence.
Employment status of the participant	It consisted multiple categories such as unemployed, service, business, handicraft, agriculture, farm/fishing and day labour, etc.
Gravida	The total number of all pregnancies reported by a woman had in her lifetime.
Availability of calendar at home	Calendar was available at home during interview.
Purpose of using calendar	We asked the study participants about the purposes of using calendar. Multiple responses were included such as- calculate days and dates, track the dates of menstrual period, remember the special/festival day (Hat day/Eid/Puja/selling certain goods), remember the date of tuition fee of children, remember the loan instalment day, child's date of birth and track lunar date.
Wealth Index	The wealth index was calculated using easy-to-collect data on a household's ownership of selected assets, including main source of drinking water, boiled water prior to drink, water source for cooking and hand washing, toilet facility, cooking fuel, main construction material of the roof, main material of the exterior walls, main material of the floor, rooms used for sleeping, ownership of farm animals, own household, own agricultural land. Besides these, ownership of durable goods such as- radio, television, mobile phone, refrigerator, shallow machine, computer; ownership of transport such as- bus, truck, rickshaw, van, motorcycle. Wealth index was a categorical variable which categorized into poorer, poor, middle, rich and richest.

Statistical analysis

Quantitative analysis was performed using the statistical software package STATA, version 13. Descriptive analyses were conducted to describe characteristics of the study participants and also for calendar literacy and LMP recall. Mean and frequencies were computed for continuous and categorical variables, respectively, considering 95% confidence interval. Logistic regression analysis was performed to examine the association between calendar literacy and different covariates. Similarly, to see the effect of covariates on LMP recall, we executed logistic regression. Multinomial logistic regression was used to examine the association between calendar literacy and LMP recall. Both regression analyses were controlled for socio-demographic characteristics including age, place of residence, education level, employment status and economic status since these factors might influence the calendar literacy and recall of LMP. All statistically significant results were reported at p value <0.05.

Principal Component Analysis method was applied to conduct wealth index analysis.[32 33] The wealth index is a composite measure of a household's cumulative living standard. Information on the wealth index is based on the data collected through household questionnaire. Each household is assigned a standardized score for each asset and collective score differs from depending on whether the household owned the assets or not. These scores are summed up by the availability of household assets and individuals are ranked according to the total score of the household in which they resided. The resulting asset scores are standardized in relation to a standard normal distribution with a mean of zero and a standard deviation of one. The sample is then divided into population quintiles - five groups with the same number of individuals in each. These standardized scores are then used to create the break points that defined wealth quintiles as: poorer, poor, middle, rich and richest.

Results

Socio-demographic characteristics of the study participants

Table 1 shows the socio-demographic characteristics of the study participants. During the survey, most of the participants (62%) belonged to the age group of 20 to 29 years followed by 22%, who were below 20 years of age. Overall 80% women had education of more than 5 years and 51% had completed 8 years of schooling and above. Among the participants, 95% of women were

For peer review only - http://bmjopen.bmj.com/site/about/guidelines.xhtml

unemployed. Fifty percent of the participants mentioned that they had experienced two pregnancies (median 2).

Table 1 Socio demographic characteristics of the survey participants

**Traits**	**Total n =2731 (%)**
**Age in years**	
≤19	609(22.3)
20-29	1693(62.0)
≥30	429(15.7)
**Completed years of schooling**	
<5	541 (19.8)
5-7	796 (29.2)
≥ 8	1394 (51.0)
**Residence**	
Urban	915 (33.5)
Rural	1816 (66.5)
**Employment status of the participant**	
Unemployed	2583(94.6)
Employed*	148(5.4)
**Gravida**	
1	1038 (38.0)
≥2	1693 (62.0)
**Median no. of pregnancies (Gravida)**	2
**Wealth index**	
Poorer	547(20.0)
Poor	546(20.0)
Middle	546(20.0)
Rich	546(20.0)
Richest	546(20.0)

*refers Service/Business/Handicraft/Agriculture/Farm/Fishing, and Day labour, etc.

For peer review only

Calendar literacy, use and availability

Participants who could mention the day, month and year instantly without seeing the calendar

The participants were asked to mention the name of the present day, month and year (on the day of interview) instantly according to the Bengali and the English calendar. Figure 1 shows almost all the participants (97%) were able to mention the name of days as per Bengali calendar which was almost same in both rural and urban areas. On the other hand, 45% participants were able to mention the name of days from the English calendar while participants from urban areas were better than rural areas (56% Vs 40%). Majority of the urban women (77%) were able to mention the name of the month from the English calendar rather than the name of month in the Bengali calendar (44%). More rural women (64%) mentioned the name of the months from the Bengali calendar compared to the English calendar (54%). However, name of the year was mentioned accurately based on the English calendar (urban 85% Vs rural 72%) whereas only few participants could name of the year from the Bengali calendar (urban 7% Vs rural 6%). In case of remembering date, majority participants both in urban and rural areas (78% and 58%) were able to correctly mention the present date based on the English calendar while only 12% of total participants mentioned the date from the Bengali calendar.

Ability and purpose of using calendar

"Table 2 Ability and purpose of using calendar" shows that, more than half of the women (61%) marked the date correctly from the English calendar whereas 16% marked from the Bengali calendar and only a few of women (4%) marked from the Hijri calendar. About 63% women could mark correctly from any of these three calendars (Bengali, English and Hijri) which we considered as calendar literacy. More than half of the participants (58%) had calendar at their home. About 44% of women reported to use it for calculating days and dates whereas only 10% women used it to track their menstrual cycle.

For peer review only - http://bmjopen.bmj.com/site/about/guidelines.xhtml

Table 2 Ability and purpose of using calendar

Traits	Total n=2731(%)
Could mark date from the Bengali calendar	448(16.4)
Could mark date from the English calendar	1668(61.1)
Could mark date from the Hijri calendar	114(4.2)
Could mark date from any of these three calendars	1730(63.4)
**Availability of calendar at home**	
Yes	1594(58.4)
No	1137(41.6)
**Purpose of using calendar***	
To calculate the days and dates	1204(44.1)
To track the dates of menstrual period	268(9.8)
To remember the special/festival day (Hat day/Eid/Puja/Selling certain goods)	364(13.3)
To remember the date of tuition fee of children¨	85(3.1)
To remember the loan instalment day	61(2.2)
Others*[1]	39(1.4)
* refers multiple responses<br>*[1] refers child date of birth, calculate lunar date, etc.	

Calendar literacy and its associated factors

Association between calendar literacy and socio-demographic characteristics

"Supplementary Table 1 Association between calendar literacy and socio-demographic characteristics of the study participants" indicates majority participants (67%) from age group 20-29 were calendar literate. The women who were employed had more (81%) calendar literacy. The women who had completed 8 or more years of schooling, most of them (89%) were calendar literate. The women who did not complete 5 years of schooling, 90% of them were calendar illiterate.

Educational status of calendar literate women

In Figure 2 we explored the relationship between calendar literacy and educational background of the study participants. The women who could mark the correct date in the calendar, most of them

For peer review only - http://bmjopen.bmj.com/site/about/guidelines.xhtml

(72%) had completed 8 or more years of schooling. Only a few (3%) of them had no schooling or did not complete more than 4 years of schooling.

Factors associated with calendar literacy

"Supplementary Table 2 results from logistics regression on calendar literacy" presents that almost all the indicators were crudely associated with higher prevalence of calendar literate women. After adjustment, the odds ratio of calendar literate women who completed 8 or more years of schooling was 34.5 times (adj. OR 34.5, 95% CI: 24.00, 49.57) higher than the women who did not complete 5 years of schooling. The women who belonged to the top two wealth quintiles (i.e. rich adj. OR 2.75, 95% CI: 1.83, 4.11 and richest adj. OR 3.15, 95% CI: 1.88, 5.29) were 2.75 and 3.15 times respectively more calendar literate than the poorer women.

LMP recall

"Table 3 association between LMP recall and socio-demographic characteristics" presents more than half (53%) of the participants could recall their recent LMP dates.

About 59% women who recalled their recent LMP dates belonged to the age group between13-19 years. Significant statistical ($p<0.01$) association was found between education and the LMP recall. The women who had completed 8 or more years of schooling, most of them (60%) could recall the LMP date. The women who were calendar literate, about 60% of them could recall their recent LMPs and there was a significant (p-value<0.01) association between calendar literacy and LMP recall.

Table 3 Association between LMP recall and socio-demographic characteristics

Traits	Recall recent LMP		P-value
	**Yes 1446 (%)**	**No 1285 (%)**	
**Age in years**			
≤19	359(58.9%)	250(41.1%)	
20-29	899(53.1%)	794(46.9%)	<0.001
30+	188(43.8%)	241(56.2%)	

For peer review only - http://bmjopen.bmj.com/site/about/guidelines.xhtml

**Completed years of schooling**			
<5	201(37.2%)	340(62.8%)	
5-7	406(51.0%)	390(49.0%)	<0.001
≥ 8	839(60.2%)	555(39.8%)	
**Residence**			
Rural	948(52.2%)	868(47.8%)	<0.001
Urban	498(54.4%)	417(45.6%)	
**Employment status of the participant**			
Unemployed	1365(52.8%)	1218(47.2%)	<0.001
Employed*1	81(54.7%)	67(45.3%)	
**Gravida**			
1	669(64.5%)	369(35.5%)	<0.001
≥2	777(45.9%)	916(54.1%)	
**Wealth index**			
Poorer	235(43.0%)	312(57.0%)	
Poor	288(52.7%)	258(47.3%)	
Middle	292(53.5%)	254(46.5%)	<0.001
Rich	308(56.4%)	238(43.6%)	
Richest	323(59.2%)	223(40.8%)	
**Availability of calendar at home**			
Yes	893(56.0%)	701(44.0%)	<0.001
No	553(48.6%)	584(51.4%)	
**Purpose of using calendar*2**			
To calculate the days and dates	703(58.4%)	501(41.6%)	
To track the dates of menstrual period	168(62.7%)	100(37.3%)	
To remember the special/festival day (Hat day/ Eid/Puja/Selling certain goods)	288(65.5%)	152(34.5%)	0.042
To remember the date of tuition fee of children¨	55(57.9%)	40(42.1%)	
To remember the loan instalment day	33(54.1%)	28(45.9%)	
Others*3	22(56.4%)	17(43.6%)	
**Calendar literacy**			
Literate	1034(59.8%)	696(40.2%)	<0.001
Illiterate	412(41.2%)	589(58.8%)	

**Track LMP date**			
Written	1394(53.9%)	1194(46.1%)	<0.001
Unwritten	52(36.4%)	91(63.6%)	
**Total**	1446 (53.0%)	1285 (47.0%)	
***1** refers Service/Business/Handicraft/Agriculture/Farm/Fishing, and Day labor, etc.<br>*2 refers multiple response<br>*3 refers child's date of birth, calculate lunar date, etc.			

LMP recall and its associated factors

"Table 4 Crude and adjusted OR for the factors associated with LMP recall" presents that almost all the indicators were crudely associated with the higher prevalence of recalling LMP. After adjustment, the odds ratio of LMP recall for the women who completed 8 or more years of schooling was 1.39 times (adj. OR 1.39, 95% CI: 1.05, 1.84) higher than the women who did not complete 5 years of schooling. Similarly, the richest women were more likely to recall recent LMP than the poorer women (rich: adj. OR 1.51, 95% CI: 1.12, 2.03 and richest: 1.61, 95% CI: 1.13, 2.30). The odds ratio of LMP recall for calendar literate women was 1.59 times (adj. OR 1.59, 95% CI: 1.28, 1.98) higher than the calendar illiterate women.

For peer review

Table 4 Crude and adjusted OR for the factors associated with LMP recall

**Covariates**	cOR (95% CI lower to upper)	aOR	(95% CI lower to upper)	P value
**Constant**		0.41	(0.31 to 0.55)	<0.01*
**Age group of women**				
≤19 years	1.84 (1.43 to 2.36)	1.11	(0.82 to 1.5)	0.52
20 to 29 years	1.45 (1.17 to 1.8)	1.10	(0.88 to 1.38)	0.41
≥ 30 years	1.0	1.0		
**Completed years of schooling**				
<5	1.0	1.0		
5-7	1.76 (1.41 to 2.2)	1.26	(0.98 to 1.61)	0.07
≥8	2.56 (2.08 to 3.14)	1.39	(1.05 to 1.84)	0.02*
**Residence**				
Rural	1.0	1.0		

For peer review only - http://bmjopen.bmj.com/site/about/guidelines.xhtml

Urban	1.09 (0.93 to 1.28)	0.80	(0.63 to 1)	0.05
**Employment status of the participant**				
Unemployed	1.0	1.0		
Employed*	1.08 (0.77 to 1.5)	0.85	(0.6 to 1.21)	0.37
**Gravida**				
1	2.14 (1.82 to 2.51)	1.88	(1.55 to 2.29)	<0.01*
≥2	1.0	1.0		
**Wealth index**				
Poorer	1.0	1.0		
Poor	1.48 (1.17 to 1.88)	1.28	(1 to 1.64)	0.05*
Middle	1.53 (1.2 to 1.94)	1.28	(0.99 to 1.66)	0.06
Rich	1.72 (1.35 to 2.18)	1.51	(1.12 to 2.03)	<0.01*
Richest	1.92 (1.51 to 2.45)	1.61	(1.13 to 2.3)	<0.01*
**Availability of calendar at home**				
No	1.0	1.0		
Yes	1.35 (1.15 to 1.57)	0.95	(0.74 to 1.21)	0.66
**Using calendar to calculate days and dates**				
No	1.0	1.0		
Yes	1.48 (1.27 to 1.72)	0.94	(0.73 to 1.23)	0.67
**Using calendar to track the dates of menstrual period**				
No	1.0	1.0		
Yes	1.56 (1.2 to 2.02)	1.23	(0.93 to 1.63)	0.15
**Calendar literacy**				
Literate	2.12 (1.81 to 2.49)	1.59	(1.28 to 1.98)	<0.01*
Illiterate	1.0	1.0		

Significant p value ≤0.05.

aOR, adjusted OR; cOR, crude OR.

CI – Confidence interval

*refers Service/Business/Handicraft/Agriculture/Farm/Fishing, and Day labour, etc.

Model adjusted for variables such as woman's age, completed years of schooling, residence, employment status, gravida, wealth index, availability of calendar at home, purpose of using calendar, tracking LMP and calendar literacy.

Discussion

This study explored the prevalence of LMP recall and calendar literacy, how women track their LMP dates, use and availability of calendar and the factors associated with calendar literacy and LMP recall. The study found that the calendar literacy rate was much higher in urban areas than that of rural areas. Most of the participants could mention the dates in English over Bengali. However, names of the days were more commonly answered in Bengali. Approximately, more than half of the women were able to mark any type of calendar correctly. Furthermore, among the women who were using calendar for menstrual tracking, most of them were calendar literate. Our study also found significant association between LMP recall and education level. Women with completed 8 or more years of schooling were more able to recall their LMP than the women who did not complete 5 years of schooling. We further found that women who were calendar literate can recall LMP 1.59 times higher than those who were calendar illiterate. We also found association between LMP recall and economic status where rich women were more likely to recall LMP more accurately than the poorer women.

To our best knowledge, this is the first study of its kind reporting the prevalence of calendar literacy and the factors associated with calendar literacy and how calendar literacy contributes to LMP recall in Bangladesh. We could not compare our major findings since there has not yet been any other study published. At the same time, we did not find any systematic review or meta-analysis in Bangladesh reporting the prevalence of either calendar literacy or LMP recall.

A study about using home calendar was done in Bangladesh in 2016. The study examined the significance of LMP recall to measure the gestational age by using home calendar and explored how it differed from ultrasound result. That study reported to validate the use of prospectively collected LMP for estimating gestational age in a low-income and rural setting of Bangladesh.[34]

For peer review only - http://bmjopen.bmj.com/site/about/guidelines.xhtml

Another study conducted in 2005 compared the recalling dates of LMPs with prospectively recorded dates. The study found that most women were able to recall their LMP accurately. But once the length of recall went beyond three weeks, women generally overestimated the dates.[18]

A study conducted in Bangladesh revealed that mother's higher education level and previous experience of preterm birth is associated with better accuracy of LMP recall.[34] LMP can predict gestational age well if cycle characteristics and the date of onset of the last menstrual bleed can be clearly established, yet this has been proven difficult in many low and middle income countries.[35] Another study conducted in rural Bangladesh also conveyed that in some settings, LMP dates are greatly considered to estimate gestational age accurately.[36] Studies also presented evidence that it is a reliable way to calculate gestational age where one study had precision level of 86-90% for LMP recall's accuracy and reliability.[14 37] Despite the limitations, studies conducted in different geographical settings such as Bangladesh and Guatemala have recommended LMP to be the chosen method for determining gestational age in low-resource settings.[12 38 39]

In many studies related to preterm birth, fetal growth restriction and maternal health, we found dependency and consideration of LMP date and prioritizing it to determine gestational age.[15 40-42]

Several studies we discussed used LMP recall method to identify pregnancy and utilized paper-based calendar as a tool to get the accurate LMP recall and recruited pregnant women immediately after pregnancy confirmation for their surveillance or interventions.[12 14 15 18 34-42] Our cross sectional survey only explored the use of calendar, calendar literacy, LMP recall and its associated factor in the area where there is no such paper-based calendar intervention. In our country context women usually remember LMP dates through unwritten way. This study found that only 10% women used calendar for tracking LMP.

However, in the modern era, everyone seems to be connected to the digital world, with more and more people using calendars inbuilt in their phone, their email, or various other options. In many places like homes and businesses still use at least one printed paper calendar.[43] This study also found that 58% households owned a printed calendar and 93% owned (according to our household asset calculation) at least one cell phone. Printed calendar provides an easy way to see the date

For peer review only - http://bmjopen.bmj.com/site/about/guidelines.xhtml

without having to log in and access a tiny screen on the smart phone. So, using printed calendar to track LMP is still easy and relevant for women even in the age of digital technology where electronic calendar is available in the cell phone.

In Bangladesh, tracking one's menstrual cycle using a calendar is a concept which is yet to become common and practiced. If women are calendar literate, they will be able to have a clear idea about their menstrual cycle, monitor their cycle and detect any menstrual abnormalities. In addition to these, they will be able to calculate the gestational age properly and identify pregnancy at an earlier stage. Early identification of pregnancy can lead to proper and timely utilization of ANC. This would prevent complications during pregnancy and delivery for the mothers and the babies thus to improve birth outcomes. Another study affirms that woman who received adequate number of ANC at the right times by a skilled attendant was more likely to have the delivery by a skilled attendant which contributes significantly to improve maternal and child health and smoothen the path to achieve SDG goals.[44]

Since this study was conducted in the Northern part of Bangladesh where ANC coverage is higher, literacy rate is moderate and human resource gaps are low; hence, the findings are subject to compare contextually. Analysis was done depending on self-reported information without having a strong surveillance system, therefore, scope of over or under reporting may exist. Because of self-reported data, LMP recall may vary. There may be some other confounding factors (e.g. lack of awareness about importance of gestational age, irregular menstrual cycle, etc.) that were not explored in this study.

Conclusion and recommendation

Recalling LMP accurately is very crucial for maternal health as it helps to calculate the gestational age accurately. It also helps to detect pregnancy early which can have influence on early initiation of pregnancy care. We found significant relationship between calendar literacy and LMP recall. Though the calendar literacy rate and the availability of calendar at home are good, however; practice of LMP tracking using calendar is very low. Besides calendar literacy, factors like education, place of residence and socio-economic status have a great influence on LMP recall too. Mounting all the relevant evidences, we summed up; calendar literacy is an opportunity to

For peer review only - http://bmjopen.bmj.com/site/about/guidelines.xhtml

introduce a simple tool or paper-based calendar in any MNCH programmes at communities where ultrasound not available to ensure accurate LMP recall for early pregnancy registration and timely ANC coverage for improving maternal health outcomes in Bangladesh.

Authors' contributions

BKS, MR, DKM, MKM, AA and AR conceived and designed the experiments. BKS, MR, TR2, JJK, MH and MSM performed the experiments. BKS, TR1, TR2, FA, MH, SNM, AA and AR were involved in analysing data. BKS, MR, TR2, JJK, TR1, MH, FA, MSM and FR contributed to developing the tools. BKS, MR, TR1, TR2, FR, SNM, DKM, MKM, AA and AR wrote the paper.

Competing interests

The authors declare that they have no competing interests.

Funding statement

This research protocol was funded by Bill & Melinda Gates Foundation. The awarded BMGF grant number is OPP1146943.

Data availability statement

Data are available upon reasonable request. Unpublished data are available in accordance with icddr,b's data sharing policy. Requests should be addressed to Ms. Armana Ahmend, Head, Research Administration, icddr,b; aahmed@icddrb.org. More information can be found here-https://eur01.safelinks.protection.outlook.com/?url=https%3A%2F%2Fwww.icddrb.org%2Fdmdocuments%2Ficddrb%2520Data%2520Access%2520Policy.pdf&data=02%7C01%7Canne.laterra%40care.org%7C73604d2a11bc4750c21608d75e12da16%7Ce83233b748134ff5893ff60f400bfcba%7C0%7C1%7C637081307376649360&sdata=fLeMIOsL2d%2FnkdoDykAoa9kmPxhn3hnJutjwEkOScI4%3D&reserved=0.

For peer review only - http://bmjopen.bmj.com/site/about/guidelines.xhtml

Patient and Public Involvement

Patients or the public were not involved in the design, or conduct, or reporting, or dissemination plans of this study

Acknowledgement

This research protocol was funded by Bill & Melinda Gates Foundation. The awarded BMGF grant number is OPP1146943. icddr,b acknowledges with gratitude the commitment of Bill & Melinda Gates Foundation to its research efforts. We are grateful to our study participants for their spontaneous participation and sincere commitment to fulfil the research endeavour. icddr,b is also grateful to the Governments of Bangladesh, Canada, Sweden and the UK for providing core/ unrestricted support.

Figure legends

"Figure 1: Women who could mention current date"

"Figure 2: Educational status of calendar literate women"

References

1. Alkema L, Chou D, Hogan D, et al. Global, regional, and national levels and trends in maternal mortality between 1990 and 2015, with scenario-based projections to 2030: a systematic analysis by the UN Maternal Mortality Estimation Inter-Agency Group. *The Lancet* 2016;387(10017):462-74.

2. Wanjira C, Mwangi M, Mathenge E, et al. Delivery practices and associated factors among mothers seeking child welfare services in selected health facilities in Nyandarua South District, Kenya. *BMC public health* 2011;11(1):360.

3. Streatfield PK, El Arifeen S, Al-Sabir A, et al. Bangladesh maternal mortality and health care survey 2010: summary of key findings and implications. *Dhaka, Bangladesh: National Institute of Population Research and Training (NIPORT)* 2011

4. National Institute of Population Research Training , International Centre for Diarrhoeal Disease Research Bangladesh , MEASURE Evaluation. Bangladesh Maternal Mortality and Health

For peer review only - http://bmjopen.bmj.com/site/about/guidelines.xhtml

Care Survey 2016: Preliminary Report: NIPORT, icddr, b, and MEASURE Evaluation Dhaka, Bangladesh, and Chapel Hill, 2017.

5. Dunson DB, Colombo B, Baird DD. Changes with age in the level and duration of fertility in the menstrual cycle. *Human reproduction* 2002;17(5):1399-403.

6. Sohda S, Suzuki K, Igari I. Relationship between the menstrual cycle and timing of ovulation revealed by new protocols: analysis of data from a self-tracking health app. *Journal of medical Internet research* 2017;19(11):e391.

7. Stanford JB, White Jr GL, Hatasaka H. Timing intercourse to achieve pregnancy: current evidence. *Obstetrics & Gynecology* 2002;100(6):1333-41.

8. Wilcox AJ, Weinberg CR, Baird DD. Timing of sexual intercourse in relation to ovulation—effects on the probability of conception, survival of the pregnancy, and sex of the baby. *New England Journal of Medicine* 1995;333(23):1517-21.

9. Tunon K, Eik-Nes S, Grøttum P. A comparison between ultrasound and a reliable last menstrual period as predictors of the day of delivery in 15 000 examinations. *Ultrasound in Obstetrics and Gynecology: The Official Journal of the International Society of Ultrasound in Obstetrics and Gynecology* 1996;8(3):178-85.

10. Ghani A, Nahar A, Sultana N, et al. Prediction of Gestational age by Last Menstrual Period (LMP) in Comparison to Ultrasonography (USG). *Journal of Shaheed Suhrawardy Medical College* 2014;6(2):82-86.

11. World Health Organization. Home-based maternal records: guidelines for development, adaptation and evaluation: World Health Organization 1994.

12. Salam SS, Ali NB, Rahman AE, et al. Study protocol of a 4-parallel arm, superiority, community based cluster randomized controlled trial comparing paper and e-platform based interventions to improve accuracy of recall of last menstrual period (LMP) dates in rural Bangladesh. *BMC public health* 2018;18(1):1-8.

13. Macaulay S, Buchmann EJ, Dunger DB, et al. Reliability and validity of last menstrual period for gestational age estimation in a low-to-middle-income setting. *Journal of Obstetrics and Gynaecology Research* 2019;45(1):217-25.

14. Rosenberg RE, Ahmed ANU, Ahmed S, et al. Determining gestational age in a low-resource setting: validity of last menstrual period. *Journal of health, population, and nutrition* 2009;27(3):332.

For peer review only - http://bmjopen.bmj.com/site/about/guidelines.xhtml

15. Karl S, Suen CSLW, Unger HW, et al. Preterm or not–an evaluation of estimates of gestational age in a cohort of women from rural Papua New Guinea. *PloS one* 2015;10(5):e0124286.

16. Van Oppenraaij R, Eilers P, Willemsen S, et al. Determinants of number-specific recall error of last menstrual period: a retrospective cohort study. *BJOG: An International Journal of Obstetrics & Gynaecology* 2015;122(6):835-41.

17. Waller DK, Spears W, Gu Y, et al. Assessing number-specific error in the recall of onset of last menstrual period. *Paediatric and Perinatal Epidemiology* 2000;14(3):263-67.

18. Wegienka G, Baird DD. A comparison of recalled date of last menstrual period with prospectively recorded dates. *Journal of Women's Health* 2005;14(3):248-52.

19. Kamana K, Shakya S, Zhang H. Gestational diabetes mellitus and macrosomia: a literature review. *Annals of Nutrition and Metabolism* 2015;66(Suppl. 2):14-20.

20. Cnattingius S, Villamor E, Lagerros Y, et al. High birth weight and obesity—a vicious circle across generations. *International journal of obesity* 2012;36(10):1320-24.

21. Salahuddin M. Predictors of severe obesity in low-income, predominantly Hispanic/Latino children: the Texas Childhood Obesity Research Demonstration Study. *Preventing chronic disease* 2017;14

22. Poma PA. Early detection of pregnancy. *Journal of the National Medical Association* 1984;76(3):305.

23. Bangladesh Bureau of Statistics, Statistics and Informatics Division, Ministry of Planning. Bangladesh Population and Housing Census 2011, Community report, Dinajpur: Bangladesh Bureau of Statistics (BBS), Statistics and Informatics Division (SID), Ministry of Planning, 2014.

24. Bangladesh Bureau of Statistics, Statistics and Informatics Division, Ministry of Planning. Population and Housing Census, Community report, Nilphamari: Bangladesh Bureau of Statistics (BBS), Statistics and Informatics Division (SID), Ministry of Planning, 2013.

25. Bangladesh Bureau of Statistics, Statistics and Informatics Division, Ministry of Planning. District Statistics 2011, Rajshahi: Bangladesh Bureau of Statistics (BBS), Statistics and Informatics Division (SID), Ministry of Planning, 2013.

26. Bangladesh Bureau of Statistics, Statistics and Informatics Division, Ministry of Planning. Population & Housing Census 2011: Bangladesh Bureau of Statistics (BBS), Statistics and Informatics Division (SID) and Ministry of Planning, 2015.

For peer review only - http://bmjopen.bmj.com/site/about/guidelines.xhtml

27. MEASURE Evaluation, ICDDR B. Bangladesh District level socio-demographic and health care utilization indicators: National Institute of Population Research and Training (NIPORT), MEASURE Evaluation, UNC-CH, USA and ICDDR,B, 2011.

28. Human Resource management, Ministry of Health and Family Welfare. HRH Data Sheet 2014: Human Resource management (HRM), Ministry of Health and Family Welfare (MOHFW), Bangladesh Secretariat, Dhaka, 2014.

29. Hayes R, Bennett S. Simple sample size calculation for cluster-randomized trials. *International journal of epidemiology* 1999;28(2):319-26.

30. Bostoen K, Chalabi Z. Optimization of household survey sampling without sample frames. *International journal of epidemiology* 2006;35(3):751-55.

31. Myatt M. A short guide to undertaking surveys using the Simple Spatial Survey Method (S3M)2012.

32. Vyass S, Kumaranayake L. Constructing socioeconomic status indexes: how to use principal component analysis. *Health Policy Plan* 2006;21(6):459-68.

33. Wold S, Esbensen K, Geladi P. Principal component analysis. *Chemometrics and intelligent laboratory systems* 1987;2(1-3):37-52.

34. Gernand AD, Paul RR, Ullah B, et al. A home calendar and recall method of last menstrual period for estimating gestational age in rural Bangladesh: a validation study. *Journal of Health, Population and Nutrition* 2016;35(1):34.

35. Unger H, Thriemer K, Ley B, et al. The assessment of gestational age: a comparison of different methods from a malaria pregnancy cohort in sub-Saharan Africa. *BMC pregnancy and childbirth* 2019;19(1):1-9.

36. Shah R, Mullany LC, Darmstadt GL, et al. Incidence and risk factors of preterm birth in a rural Bangladeshi cohort. *BMC pediatrics* 2014;14(1):112.

37. Ellertson C, Elul B, Ambardekar S, et al. Accuracy of assessment of pregnancy duration by women seeking early abortions. *The Lancet* 2000;355(9207):877-81.

38. Persson LÅ, Arifeen S, Ekström E-C, et al. Effects of prenatal micronutrient and early food supplementation on maternal hemoglobin, birth weight, and infant mortality among children in Bangladesh: the MINIMat randomized trial. *Jama* 2012;307(19):2050-59.

For peer review only - http://bmjopen.bmj.com/site/about/guidelines.xhtml

39. Neufeld LM, Haas JD, Grajéda R, et al. Last menstrual period provides the best estimate of gestation length for women in rural Guatemala. *Paediatric and perinatal epidemiology* 2006;20(4):290-98.

40. Rashid H, Ma E, Ferdous F, et al. First-trimester fetal growth restriction and the occurrence of miscarriage in rural Bangladesh: A prospective cohort study. *PloS one* 2017;12(7):e0181967.

41. Baqui A, Rosen H, Lee A, et al. Preterm birth and neonatal mortality in a rural Bangladeshi cohort: implications for health programs. *Journal of Perinatology* 2013;33(12):977-81.

42. Lee AC, Mullany LC, Ladhani K, et al. Validity of newborn clinical assessment to determine gestational age in Bangladesh. *Pediatrics* 2016;138(1):e20153303.

43. VIIKING. Why Printed Calendars are Still Relevant in the Digital Age [cited 2020 08/07/2020]. Available from: https://mrshirt.com/printed-calendars-are-still-relevant.

44. Al Kibria GM, Ghosh S, Hossen S, et al. Factors affecting deliveries attended by skilled birth attendants in Bangladesh. *Maternal Health, Neonatology and Perinatology* 2017;3(1):7.

For peer review only

For peer review only - http://bmjopen.bmj.com/site/about/guidelines.xhtml

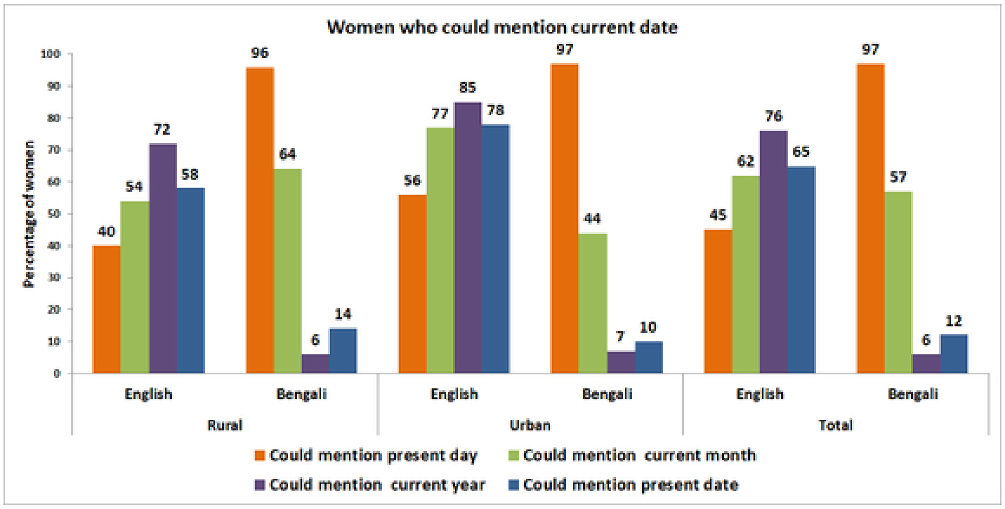

Figure 1: Women who could mention current date

48x24mm (300 x 300 DPI)

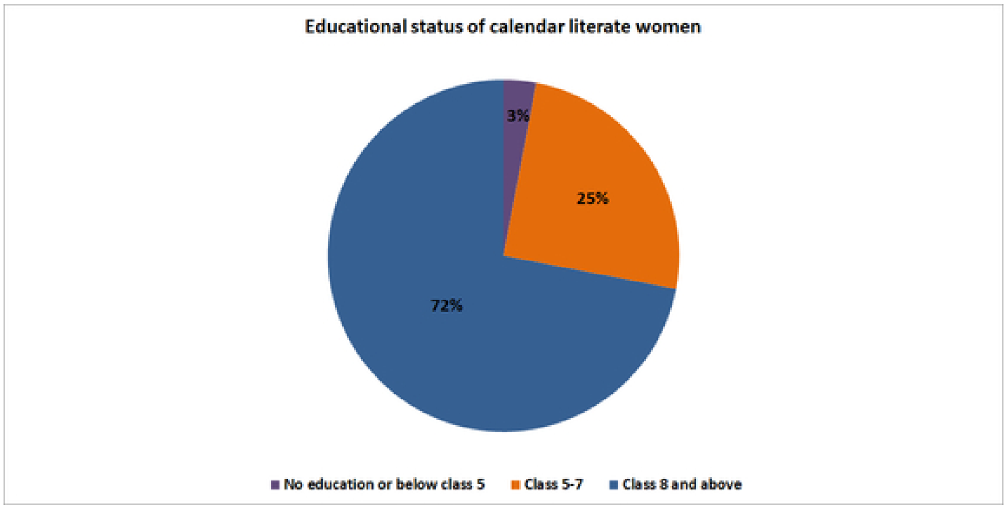

Figure 2: Educational status of calendar literate women

48x24mm (300 x 300 DPI)

Supplementary Table 1 Association between calendar literacy and socio-demographic characteristics of the study participants

Traits	Calendar literacy: Literate 1730 (%)	Calendar literacy: Illiterate 1001 (%)	P-value
**Age in years**			
≤19	362(59.4)	247 (40.6)	
20-29	1135(67.0)	558 (33.0)	<0.001
≥30	233(54.3)	196 (45.7)	
**Completed years of schooling**			
<5	52 (9.6)	489 (90.4)	
5-7	437 (54.9)	359 (45.1)	<0.001
≥ 8	1241 (89.0)	153 (11.0)	
**Residence**			
Rural	1,029 (56.7)	787 (43.3)	<0.001
Urban	701 (76.6)	214 (23.4)	
**Employment status of the participant**			
Unemployed	1610 (62.3)	973 (37.7)	<0.001
Employed*1	120 (81.1)	28 (18.9)	
**Gravida**			
1	751 (72.4)	287 (27.7)	<0.001
≥2	979 (57.8)	714 (42.2)	
**Wealth index**			
Poorer	355(64.0)	192(35.1)	
Poor	251(46.0)	295(54.0)	
Middle	213(39.0)	333(61.0)	<0.001
Rich	123(22.5)	423(77.5)	
Richest	59(10.8)	487(89.2)	
**Availability of calendar at home**			
Yes	1244(78.0)	350(22.0)	<0.001
No	486(42.7)	651(57.3)	

Purpose of using calendar*2			
To calculate the days and dates	1085(90.1)	119(9.9)	
To track the dates of menstrual period	241(89.9)	27(10.1)	
To remember the special/festival day (Hat day/Eid/Puja/selling certain goods)	342(94.0)	22(6.0)	<0.001
To remember the date of tuition fee of children	77(90.6)	8(9.4)	
To remember the loan installment day	57(93.4)	4(6.6)	
Others*3	36(92.3)	3(7.7)	

*1 refers Service/Business/Handicraft/Agriculture/Farm/Fishing, and Day labor, etc.

*2 refers multiple responses

*3 refers child's date of birth, calculate lunar date, etc.

peer

Supplementary Table 2 Results from logistics regression on calendar literacy

Covariates	cOR (95% CI lower to upper)	aOR	(95% CI lower to upper)	P value
**Constant**		0.05	(0.03 to 0.08)	<0.01*
**Age in years**				
≤19 years	1.2 (0.96 to 1.58)	0.95	(0.61 to 1.47)	0.81
20 to 29 years	1.7 (1.4 to 2.1)	1.20	(0.84 to 1.69)	0.31
≥ 30 years	1.0	1.0		
**Completed years of schooling**				
<5	1.0	1.0		
5-7	11.4 (8.3 to 15.7)	8.35	(5.94 to 11.73)	<0.01*
≥8	76.3 (54.8 to 106.3)	34.46	(23.96 to 49.57)	<0.01*
**Residence**				
Rural	1.0	1.0		
Urban	2.5 (2.1 to 3.0)	1.20	(0.85 to 1.69)	0.30
**Employment status of the participant**				
Unemployed	1.0	1.0		
Employed*	2.6 (1.7 to 3.9)	1.83	(0.99 to 3.38)	0.05

**Gravida**				
1	1.9 (1.6 to 2.3)	1.21	(0.91 to 1.60)	0.18
≥2	1.0	1.0		
**Wealth index**				
Poorer	1.0	1.0		
Poor	2.2 (1.7 to 2.8)	1.71	1.25 to 2.34	<0.01*
Middle	2.9 (2.3 to 3.7)	1.28	(0.92 to 1.77)	0.139
Rich	6.4 (4.9 to 8.3)	2.75	(1.83 to 4.11)	<0.01*
Richest	15.3 (11.1 to 21.1)	3.15	(1.88 to 5.29)	<0.01*
**Availability of calendar at home**				
No	1.0	1.0		
Yes	4.8 (4.0 to 5.6)	0.65	(0.48 to 0.88)	<0.01*
**Purpose of using calendar**				
To calculate the days and dates/ installation day	12.5 (10.1 to 15.5)	6.41	(4.57 to 8.98)	<0.01*
To track the dates of menstrual period	5.8 (3.9 to 8.8	1.08	(0.66 to 1.76)	0.76

Significant p value ≤0.05.
aOR, adjusted OR; cOR, crude OR.
CI – Confidence interval
*****refers Service/Business/Handicraft/Agriculture/Farm/Fishing, and Day labor, etc.
Model adjusted for variables such as woman's age, completed years of schooling, residence, employment status, gravida, wealth index, availability of calendar at home, purpose of using calendar

Research Checklist

Section/Topic	Item #	Description	Reported on page #
**Title and abstract**	1	(*a*) Study design within abstract	2
		(*b*) Summary of what was done and what was found	2
**Strength and Limitation**	2	Four bullet points on strength and limitation of this Study	3
**Introduction**			
Background/rationale	3	Scientific background and rationale of this study	4-5
Objectives	4	Specific objectives of this study	5
**Materials and Methods**			
Study design and settings	5	Study design, settings of the study, data collection period	5
Sampling and study participants	6	Sampling strategy, sample size, inclusion criteria of study participants	6
Data collection	7	Survey tool development, staff recruitment and training	7-8
Data management and quality assurance	8	How the quality of data was ensured, how the data were managed	8
Ethics	9	How the research integrity and ethics ensured during the study	8-9
Variables and Measures	10	Primary and secondary outcome variables, Operational definition of primary, secondary and other independent variable	9-10
Statistical analysis	11	Descriptive statistics, multiple logistic regression, Principal Component Analysis are done for the statistical analysis	11
**Results**			
Socio-demographic characteristics of the study participants	12	Age, completed years of schooling, place of residence, employment status and wealth index of the study participants are presented	11-12
Descriptive data	13	Characteristics of study participants (e.g. Age, completed years of schooling, place of residence, employment status and wealth index)	11-12
Calendar literacy	14	Prevalence of calendar literacy of the study participants is presented	13-14
Educational status of calendar literate women	15	Distribution of educational status among calendar literate women is presented	14-15
Calendar literacy and its associated factors	16	Association between calendar literacy and socio-demographic characteristics	14
LMP recall	17	Prevalence of LMP recall of the study participants	15-17
Main results	18	(a) Association between LMP recall and socio-demographic characteristics	15-17
		(*b*) Crude and adjusted OR for the factors associated with LMP recall with the precision of 95% confidence interval. Covariates such as: age, educational status, place of residence, occupation and wealth index were adjusted	17-19
**Discussion**			
Key results	19	Summary of key results	19
Limitations	20	Limitations of the study is described	21
Interpretation	21	Overall interpretation of results considering objectives, limitations, multiplicity of analyses, results from similar studies, and other relevant evidence	19-21
Generalisability	22	Discussion about generalisability of the study results	21
**Other information**			

For peer review only

Funding	23	Funding statement of the study	22
Authors' contribution and acknowledgement	24	Authors' contribution and acknowledgement of the study described	22
Reference	25	References used for this study	23-27

For peer review only

BMJ Open

Factors associated with calendar literacy and last menstrual period (LMP) recall: a prospective programmatic implication to maternal health in Bangladesh

Journal:	*BMJ Open*
Manuscript ID	bmjopen-2020-036994.R2
Article Type:	Original research
Date Submitted by the Author:	28-Sep-2020
Complete List of Authors:	Sarker, Bidhan Krishna; International Centre for Diarrhoeal Disease Research Bangladesh, Maternal and Child Health Division<br>Rahman, Musfikur; International Centre for Diarrhoeal Disease Research Bangladesh, Maternal and Child Health Divison<br>Rahman, Tanjina ; International Centre for Diarrhoeal Disease Research Bangladesh, Maternal and Child Health Division<br>Rahman, Tawhidur; International Centre for Diarrhoeal Disease Research Bangladesh, Maternal and Child Health Division<br>Rahman, Fariya; International Centre for Diarrhoeal Disease Research Bangladesh, Maternal and Child Health Division<br>Khalil, Jubaida; International Centre for Diarrhoeal Disease Research Bangladesh, Maternal and Child Health Division<br>Hasan, Mehedi; International Centre for Diarrhoeal Disease Research Bangladesh, Maternal and Child Health Division<br>Mahfuz, Sadia; University of Western Sydney<br>Ahmmed, Faisal; International Centre for Diarrhoeal Disease Research Bangladesh, Infectious Diseases Division<br>Miah, Muhammad Salim; Shahjalal University of Science and Technology, Department of Anthropology<br>Ahmed , Anisuddin; International Centre for Diarrhoeal Disease Research Bangladesh<br>Mitra, Dipak; North South University, Public Health<br>Mridha, Malay Kanti; BRAC University James P Grant School of Public Health<br>Rahman, Anisur ; International Centre for Diarrhoeal Disease Research, Maternal and Child Health Division
**Primary Subject Heading**:	Public health
Secondary Subject Heading:	Obstetrics and gynaecology, Health policy, Health services research, Sexual health
Keywords:	Health policy < HEALTH SERVICES ADMINISTRATION & MANAGEMENT, OBSTETRICS, GYNAECOLOGY, EPIDEMIOLOGY, STATISTICS & RESEARCH METHODS, PUBLIC HEALTH

SCHOLARONE™
Manuscripts

I, the Submitting Author has the right to grant and does grant on behalf of all authors of the Work (as defined in the below author licence), an exclusive licence and/or a non-exclusive licence for contributions from authors who are: i) UK Crown employees; ii) where BMJ has agreed a CC-BY licence shall apply, and/or iii) in accordance with the terms applicable for US Federal Government officers or employees acting as part of their official duties; on a worldwide, perpetual, irrevocable, royalty-free basis to BMJ Publishing Group Ltd ("BMJ") its licensees and where the relevant Journal is co-owned by BMJ to the co-owners of the Journal, to publish the Work in this journal and any other BMJ products and to exploit all rights, as set out in our licence.

The Submitting Author accepts and understands that any supply made under these terms is made by BMJ to the Submitting Author unless you are acting as an employee on behalf of your employer or a postgraduate student of an affiliated institution which is paying any applicable article publishing charge ("APC") for Open Access articles. Where the Submitting Author wishes to make the Work available on an Open Access basis (and intends to pay the relevant APC), the terms of reuse of such Open Access shall be governed by a Creative Commons licence – details of these licences and which Creative Commons licence will apply to this Work are set out in our licence referred to above.

Other than as permitted in any relevant BMJ Author's Self Archiving Policies, I confirm this Work has not been accepted for publication elsewhere, is not being considered for publication elsewhere and does not duplicate material already published. I confirm all authors consent to publication of this Work and authorise the granting of this licence.

For peer review only

Full Title

Factors associated with calendar literacy and last menstrual period (LMP) recall: a prospective programmatic implication to maternal health in Bangladesh

Short title

Associated factors related to calendar literacy and LMP recall

# Corresponding author

Bidhan Krishna Sarker[1], Assistant Scientist, Maternal and Child Health Division, icddr,b. 68, Shaheed Tajuddin Ahmed Sarani, Mohakhali, Dhaka-1212, GPO Box-1000, Dhaka, Bangladesh, Email: bidhan@icddrb.org; bidhanksarker@gmail.com

Authors

Bidhan Krishna Sarker[1], Musfikur Rahman[1], Tanjina Rahman[1], Tawhidur Rahman[1], Fariya Rahman[1], Jubaida Jahan Khalil[1], Mehedi Hasan[1], Sadia Nishat Mahfuz[2], Faisal Ahmmed[3], Muhammad Salim Miah[4], Anisuddin Ahmed[1], Dipak Kumar Mitra[5], Malay Kanti Mridha[6], Anisur Rahman[1]

Authors and Affiliations

[1]Maternal and Child Health Division, icddr,b. 68, Shaheed Tajuddin Ahmed Sarani, Mohakhali, Dhaka-1212, GPO Box-1000, Dhaka, Bangladesh

[2]MS student, University of Western Sydney, Australia

[3]Infectious Diseases Division, icddr,b. 68, Shaheed Tajuddin Ahmed Sarani, Mohakhali, Dhaka-1212, GPO Box-1000, Dhaka, Bangladesh

[4]Lecturer, Department of Anthropology, Shahjalal University of Science & Technology, Sylhet, Bangladesh

[5] Professor and Chair, Department of Public Health, North South University, Dhaka, Bangladesh

[6]Professor and Director of Centre of Excellence for Non-Communicable Diseases and Nutrition, James P Grant School of Public Health, BRAC University, Dhaka, Bangladesh

For peer review only - http://bmjopen.bmj.com/site/about/guidelines.xhtml

Abstract

Objective: To explore the prevalence and determinants of calendar literacy and last menstrual period (LMP) recall among women in Bangladesh.

Design: Cross-sectional survey.

Settings: Two rural sub-districts and one urban area from three Northern districts of Bangladesh.

Participants: We interviewed 2,731 women who had a live birth in the last one year.

Primary and secondary outcome measures: The primary outcome variable was LMP recall, and the secondary outcome was calendar literacy.

Results: The majority of participants (65%) correctly mentioned the current date according to the English calendar while 12% mentioned according to the Bengali calendar. During the interview sessions, we used three different calendars: Bengali, English, and Hijri to assess calendar literacy. We asked women to mark the current date using the calendar on the day of the interview. Almost 61% women marked the English calendar, 16% marked the Bengali calendar and 4% marked the Hijri calendar correctly. Sixty-three percent women were found as calendar literate who marked any of the calendars. Among the participants, 58% had calendars available at their home and only 10% of women used calendars to track their LMPs. Overall, 53% women were able to recall their recent LMP. Among the calendar literate, 60% could recall their LMPs. Factors found associated with recalling LMP were: completed eight or more years of schooling (adj.OR1.39), primigravida (adj.OR1.88), the richest wealth quintile (adj.OR1.55), and calendar literacy (adj.OR1.59).

Conclusions: Despite having reasonable calendar literacy and availability, the use of calendars for tracking LMP found very low. Calendar literacy and socio-demographic characteristics were found as the key factors associated with LMP recall. Maternal, Neonatal, and Child Health programmes in low-resource settings can promote a simple tool like calendar and target the communities where ultrasound is not available to ensure accurate LMP recall for early pregnancy registration and timely antenatal care coverage.

For peer review only - http://bmjopen.bmj.com/site/about/guidelines.xhtml

Strength and Limitation of this Study

1. This is the first large-scale study of its kind in Bangladesh, reporting the prevalence of last menstrual period recall in urban and rural settings.
2. The study relied on the self-reported data, but the inbuilt quality assurance system minimized the errors and improved the study results' validity.
3. We took into account both government and non-government health service catchment areas maintaining similar population coverage and statistical rigor.
4. We conducted this study in the Northern part of Bangladesh considering higher ANC coverage, moderate literacy rate was, and low human resource gaps ; hence, the findings are subject to compare contextually.
5. Several important factors such as lack of awareness about the importance of gestational age and irregular menstrual cycle were not explored which may influence the risk estimates in this study.

For peer review only

For peer review only - http://bmjopen.bmj.com/site/about/guidelines.xhtml

Introduction

Every day about 830 women die from pregnancy or childbirth-related complications around the world. Most of these deaths occur in low-resource settings, and many of them are preventable.[1] The South Asia region alone accounts for approximately one-third of the global maternal and child deaths annually.[2] Globally, the new target set by the Sustainable Development Goals for maternal health is to reduce the mortality ratio less than 70 per 100,000 live births by 2030.[1] According to the recent national data, the maternal mortality ratio in Bangladesh remains unchanged for the last decade (196 deaths per 100,000 live births) and a considerable effort is needed to achieve the Sustainable Development Goals.[3 4]

Pregnancy is an important part of women's lives, and women should have awareness regarding pregnancy care.[5-8] Reliable information about the gestational age is crucial to ensure optimal pregnancy-care.[9 10] To detect the gestational age, recall of last menstrual period (LMP) plays an important role. Recalling of LMP is also known as the "calendar method" for predicting the next ovulation date. The calendar method is based on the recognition of cycles in the menstrual period and fertility, in which women record their menstrual cycles.[6] The World Health Organization (WHO) also recommends recalling LMP for detecting gestational age because it is a simple, and low-cost method.[11 12] The LMP method has been widely accepted and used in many low and middle-income countries for calculating the gestational age and also a suggested recall method where ultrasound facilities are not readily available.[10 13-15]

Although LMP is used in low-resource settings, including Bangladesh, it is well known that the methods have inherent errors in identifying the LMP date correctly. In most cases, inaccurate LMP recall overestimates the gestational age.[12 16] Furthermore, studies conducted in both developed and developing countries have reported that 15% to 45% of women cannot recall their LMPs accurately.[12 16-18] The study has also pointed out that the validity of gestational age calculation with LMP depends on better maternal recall of dates and cycle characteristics, which is somehow related to literacy.[14]

Accurate LMP recall helps to screen for any chance of pre-eclampsia, fetal growth restriction, and premature birth outcome.[15 16 19-21] Accurate LMP recall also enables detecting pregnancy sooner

For peer review only - http://bmjopen.bmj.com/site/about/guidelines.xhtml

and facilitates early initiation of pregnancy care. Initiation of antenatal care at the right time is crucial because the first eight weeks of gestational age are very vulnerable for mothers and fetuses.[22]

Calendar literacy can be an integral part of recalling LMP accurately. We carried out this study recognizing the knowledge gap and understanding the need to know the underneath factors that expedite the calendar literacy rate and LMP recall. Therefore, this paper aims to explore the prevalence and determinants of calendar literacy and LMP recall in urban and rural areas from three Northern districts in Bangladesh.

Materials and Methods

Study design and settings

We conducted this cross-sectional study in rural and urban areas in the Northern districts from Bangladesh. The rural sites were located in the Chirirbandar and the Saidpur sub-districts under the districts of Dinajpur and Nilphamary, respectively. The urban site was located in the city corporation of Rajshahi district. According to the population and housing census (2011), Chirirbandar consisted of 142 villages under 12 unions.[23] The total population of Chirirbandar was 292,500 of which 145,881 were females. The average size of the population of each union was 24,375. Saidpur sub-district consisted of one municipality and five unions with 39 villages.[24] The total population of Saidpur was 264,461 of which 130,724 were females. The average size of the population of each union was 27,471. Rajshahi City Corporation consisted of 30 wards.[25] The total population of the Rajshahi City Corporation was 449,756 of which 216,782 were females. The average size of the population of each ward was 14,992. According to the census data, the female literacy rate was 42% in Dinajpur and 39% for both Nilphamari and Rajshahi districts.[26] Health care utilization data suggested that at least one ANC coverage in Dinajpur, Nilphamari, and Rajshahi was 75%, 96%, and 80% respectively.[27] Another data showed a medium vacancy (21% to 30%) in terms of healthcare personnel in Dinajpur, Nilphamari, and Rajshahi districts.[28] We considered moderate literacy rate, high ANC coverage, and moderate vacancy for human resources to select the study areas.

For peer review only - http://bmjopen.bmj.com/site/about/guidelines.xhtml

We considered the area served by the government and non-government community-based health workers as a unit or cluster in rural and urban sites maintaining similar population coverage. The women from each cluster who met the inclusion criteria were invited to participate in the survey. The followings were the inclusion criteria for the study participants' enrolment: (1) had a live birth outcome in the last one year, (2) past 28 or more days after the last delivery, (3) could hear, see and speak, (4) had permanent residence in the study area, and (5) provided informed consent to interview within two days of the first contact. The data collection period was from August to November 2016. In total, 2,731 women participated in the study.

Sampling and study participants

We applied two-stage cluster sampling to select study participants in the study area. In the first stage, we randomly selected one sub-district from two districts each and ten wards (lowest administrative unit of city area) from Rajshahi City Corporation. Then, we randomly selected 6 clusters out of 12 in the Chiribandar sub-district, 6 clusters out of 12 clusters in the Saidpur sub-district, and 6 clusters from 10 clusters in Rajshahi. We used a conventional statistical method to calculate the sample size. Based on the selected process indicators and assuming the improvement rate after project implementation, we calculated the sample size for the survey. As this cross-sectional study was conducted to get a baseline status in designing an intervention focusing on the improvement of maternal and child health, we considered the proportions at baseline and end line to estimate the sample size. The sample size was calculated using the formula proposed by Hayes and Bennett.[29] Considering the proportion of taking any ANC by the first trimester $p_{0=}$0.41 (baseline), $p_{1=}$0.57 (end line), and K=0.15 (coefficient of variation between clusters); we needed the highest number of 150 mothers in each cluster, including 5% non-response rate. Considering the proportion of taking at least one ANC from medically trained provider $p_{0=}$0.49 and $p_{1=}$0.66 and K=0.15, we needed the highest number of 6 clusters per area to assess the change in the indicators those were least known at baseline. For each study area, the sample size was calculated 900; the total required sample size was 2,700 (150×6×3).

We recruited study participants through the EPI (expanded programme on immunization) method.[30 31] At first, we selected the starting point to start data collection in the selected clusters. We consulted with the community people to determine the midpoint of each cluster. We used the

For peer review only - http://bmjopen.bmj.com/site/about/guidelines.xhtml

bottle spinning method to ensure randomization. We spun the bottle at the midpoint of each cluster to pick a random direction to start searching study participants. Afterwards, we visited every household on the next door basis as per bottle indicated direction, and interviewers identified eligible participants for interviewing. Data collection continued until the cluster's sample size was met. Though the required sample size was 150 in each cluster but in some cases, the data collection team interviewed one or two more women to cover the entire cluster. Finally, we completed 2,731 interviews from the three study areas. If any eligible woman was absent during the household visit, then data collectors tried at least two more times to interview her.

Data collection

Survey tool development

We used a semi-structured questionnaire to collect data through face-to-face interviews with women. A team of experienced investigators from icddr,b were responsible for developing this survey tool. Based on the research objectives and the expected outcome variables of the study, the research team considered the relevant indicators to design the tool. As part of designing the tool, investigators did the desk review of the relevant literature such as- Bangladesh Demographic and Health Survey, Bangladesh Maternal Mortality and Healthcare Survey, and other similar studies. After formulating the tool, we carried out a pre-test to check the design of the questionnaire how it works in practice. After addressing all the problems related to the survey tool, the data collection team started collecting data. In the survey tool, we included the basic individual and household characteristics of the study participants (age, religion, years of schooling, employment status, income, household asset, and place of residence); reproductive and obstetric information of the participants (age at first marriage, gravida, para, living children, pregnancy history, LMP date), knowledge and practice of calendar literacy (able to mention correctly Bengali and English day, month, year and date which is related to their knowledge; able to mark Bengali/English/Hijri date in the calendar which is related to their practice).

For peer review only - http://bmjopen.bmj.com/site/about/guidelines.xhtml

Staff recruitment and training

We involved a team of efficient and experienced field research assistants for data collection. We recruited field research assistants who had a minimum Bachelor's degree with at least two years of previous experience in the relevant field especially in similar positions in large-scale surveys. After recruitment, we trained the interviewers on the survey tool to collect data. During training sessions, the key persons from the research team discussed the objectives of the study so that the data collection team can easily avoid any confusion. Furthermore, we developed a training manual for the interviewers that included interviewing techniques and specific instructions for each of the data collection questionnaire and checklist.

Data management and quality assurance

A project research physician and a research investigator coordinated the data collection team. Furthermore, we divided the data collection team into 3 groups. Each group had 4 data collectors and a group leader. The group leaders were responsible for ensuring quality data regularly. They checked the completeness of every interview on the spot after the data collected through the day to day monitoring. Again, as per the monitoring plan, the group leaders, and the team coordinators re-interviewed almost one-third of the total interviewees to check the accuracy and the validity of data. We also checked relevant documents (such as calendar) during our data collection to minimize the errors. Expert programmers of the Maternal and Child Health Division of icddr,b designed a database template maintaining skipping options strictly and logically to avoid inconsistency of data. The programming team used Dot net (Version-10) software for data template design as appropriate. An expert data management team was engaged in entering all the data through an online database. The data management team simultaneously entered both the precoded and the postcoded data. For post-coding data, the research team was closely involved with the data management team.

Ethics

icddr,b has rigorous processes to meet research integrity and ethics, which is called the Institutional Review Board (IRB). icddr,b's IRB consists of four mandatory committees involving internal and external topic experts: Research Review Committee (RRC), Ethical Review Committee (ERC), Animal Experimentation Ethics Committee, and Programme Coordination Committee. icddr,b

For peer review only - http://bmjopen.bmj.com/site/about/guidelines.xhtml

attaches great importance to ensuring that all research protocols conducted by its scientists are scientifically sound and meet international ethical standards. To achieve this, in addition, to review at the centre level and by at least two external reviewers, all research protocols are subject to review by at least two of three committees: RRC, ERC (protocols involving human subjects), and the Animal Experimentation Ethics Committee (protocols involving animals).

RRC has the authority to approve, suggest modifications to, or disapprove of any proposed research protocol. Any change to an approved research protocol also needs the approval of the RRC. No research protocol can be implemented or proceed for consideration by the ERC or the Animal Experimentation Ethics Committee without the RRC's approval.

The ERC's approval is required for all protocols involving human subjects. It is an independent body committed to protecting the rights of the people who participate in research protocols conducted under the auspices of icddr,b. A subcommittee of the ERC, the Data Safety Monitoring Board periodically reviews and evaluates the accumulated study and makes recommendations to the ERC concerning the continuation, modification, or termination of the study.

We acquired both RRC and ERC approval from the IRB of icddr,b before we start collecting data. During the data collection period, we acquired written informed consent from all the participants after ensuring that none of them is exposed to minimal risk while participating in this study. Since all our participants were already married by then, we did not need to take consent for the minors from their guardians or parents as per IRB requirements.

For peer review only - http://bmjopen.bmj.com/site/about/guidelines.xhtml

Variables and Measures

All the information given by the mothers were self-reported. The primary outcome variable was LMP recall, and the secondary outcome was calendar literacy. The rest of the variables were considered as independent variables. The description of all variables is given below-

List of variables	Explanation
LMP recall (Primary outcome variable)	We considered the first day of LMP. We asked the women about the LMP date of their menstrual cycle prior to the interview date.
Calendar literacy (Secondary outcome variable)	We asked study participants to mark the current date from the three different types of calendars (Bengali, Hijri, and English). If the study participants marked it correctly, we coded "1" for the correct answer and "0" for the wrong answer. We considered women as calendar literate if they could accurately mark the current date using any of the provided calendars.
Mentioned the current day, month, year, and date	We asked women whether they could tell the name of the present day, month, year, and the date (the current day of interview)
Age in years	We considered completed years of age and categorised into the following ranges ≤19, 20-29, ≥30 years of age
Years of schooling	We considered completed years of schooling and categorised into <5, 5-7, and ≥8 years of schooling.
Place of residence	We considered interviewing the women with permanent residence at the respective study sites. We categorised place of residence into rural and urban areas.
Employment status of the participants	It consisted of multiple categories such as unemployed, service, business, handicraft, agriculture, farm/fishing, and day labour.
Gravida	The total number of all pregnancies reported by a woman that she had in her lifetime.
Availability of calendar at home	The calendar was available at home during the interview.
Purpose of using the calendar	We asked the study participants about the purposes of using a calendar. Multiple responses were included such as- calculate days and dates, track the dates of the menstrual period, remember the special/festival day (Hat day {weekly shopping day at village}/Eid/Puja/Selling certain goods), remember the date of the tuition fee of children, remember the loan installment day, child's date of birth and track lunar date.
Wealth index	The wealth index was calculated using easy-to-collect data on a household's ownership of selected assets including primary source of drinking water, boiled water prior to drinking, the water source for cooking and handwashing, toilet facility, cooking fuel, main construction material of the roof, the main material of the exterior walls, the main material of the floor, rooms used for sleeping, ownership of farm animals, own household, own agricultural land. Besides these, ownership of durable goods such as- radio, television, mobile phone, refrigerator,

For peer review only - http://bmjopen.bmj.com/site/about/guidelines.xhtml

shallow machine, computer; ownership of transport such as- bus, truck, rickshaw, van, motorcycle. The wealth index was a categorical variable that categorised into poorer, poor, middle, rich, and richest.

Statistical analysis

We performed a quantitative analysis using the statistical software package STATA, version 13. We used both descriptive and analytical approaches. The study participants' characteristics, including their calendar literacy and the capability of recall LMPs, were presented by mean, median, and proportion. To examine the association between outcomes and available covariates, we performed a logistic regression analysis. The potential confounders such as maternal age, place of residence, years of schooling, employment status, and economic condition by asset quintiles were adjusted in the regression analysis. The results were presented by odds ratio (OR) with 95% confidence intervals (CI). All statistically significant results were reported at p-value <0.05.

We generated wealth scores by using the principal component analysis method.[32 33] The wealth index is a composite measure of a household's cumulative living standard.[34] Information on the wealth index is based on the data collected through the household questionnaire. Each household was assigned a standardised score for each asset, and the collective score differs from one another depending on whether the household owned the assets or not. These scores are summed up by the availability of the household assets, and individuals are ranked according to the total score of the household. The resulting asset scores are standardised concerning a standard normal distribution with a mean of zero and a standard deviation of one. The sample is then divided into population quintiles - five groups with the same number of individuals in each. These standardised scores are then used to create the breakpoints that defined wealth quintiles as: poorer, poor, middle, rich, and richest.

For peer review only - http://bmjopen.bmj.com/site/about/guidelines.xhtml

Results

Socio-demographic characteristics of the study participants

Table 1 shows that most of the participants (62%) belonged to the age group of 20 to 29 years, followed by 22% who were below 20 years of age. Overall, 80% of women had completed five years of schooling of which 51% had completed eight or more years of schooling. Among the participants, 95% of women were unemployed. Fifty percent of the participants mentioned that they had experienced two pregnancies (median 2).

For peer review only

Table 1 Socio-demographic characteristics of the survey participants

**Traits**	**Total n =2731 (%)**
**Age in years**	
≤19	609(22.30)
20-29	1693(61.99)
≥30	429(15.71)
**Completed years of schooling**	
<5	541 (19.81)
5-7	796 (29.15)
≥ 8	1394 (51.04)
**Residence**	
Urban	915 (33.50)
Rural	1816 (66.50)
**Employment status of the participant**	
Unemployed	2583(94.58)
Employed*	148(5.42)
**Gravida**	
1	1038 (38.01)
≥2	1693 (61.99)
**Median no. of pregnancies (Gravida)**	2
**Wealth index**	
Poorer	547(20.00)
Poor	546(20.00)

For peer review only - http://bmjopen.bmj.com/site/about/guidelines.xhtml

Middle	546(20.00)
Rich	546(20.00)
Richest	546(20.00)
*refers to Service/Business/Handicraft/Agriculture/Farm/Fishing, and Day labour, etc.	

Calendar literacy, use, and availability

Participants who could mention the day, month, and year instantly without seeing the calendar

We asked the participants to mention the name of the current day, month, and year (on the day of interview) instantly according to the Bengali and the English calendar. Figure 1 shows almost all the participants (97%) were able to mention the name of the days as per the Bengali calendar, which was almost the same in both rural and urban areas. On the other hand, 45% of participants were able to mention the name of days from the English calendar while participants from urban areas could mention better than their rural counterpart (56% Vs 40%). The majority of the urban women (77%) were able to mention the name of the month from the English calendar rather than the name of a month in the Bengali calendar (44%). More rural women (64%) mentioned the name of the months from the Bengali calendar compared to the English calendar (54%). However, the name of the year was mentioned accurately based on the English calendar (urban 85% Vs rural 72%) whereas only a few participants could name the year from the Bengali calendar (urban 7% Vs rural 6%). In case of remembering date, majority participants both in urban and rural areas (78% and 58%) were able to correctly mention the present date based on the English calendar while only 12% of the total participants mentioned the date from the Bengali calendar.

Capability and purpose of using a calendar

Table 2 shows that more than half of the women (61%) marked the date correctly from the English calendar whereas 16% marked from the Bengali calendar and only a few of women (4%) marked from the Hijri calendar. About 63% of women could mark correctly from any of these three calendars (Bengali, English, and Hijri), which we considered as calendar literacy. More than half of the participants (58%) had a calendar at their home. About 44% of women reported using it for calculating days and dates, whereas only 10% of women used it to track their menstrual cycle.

For peer review only - http://bmjopen.bmj.com/site/about/guidelines.xhtml

Table 2 Capability and purpose of using a calendar

Traits	Total n=2731(%)
Could mark date from the Bengali calendar	448(16.40)
Could mark date from the English calendar	1668(61.08)
Could mark date from the Hijri calendar	114(4.17)
Could mark date from any of these three calendars	1730(63.35)
**Availability of calendar at home**	
Yes	1594(58.37)
No	1137(41.63)
**Purpose of using calendar***	
To calculate the days and dates	1204(44.09)
To track the dates of the menstrual period	268(9.81)
To remember the special/festival day (Hat day/Eid/Puja/Selling certain goods)	364(13.33)
To remember the date of the tuition fee of children	85(3.11)
To remember the loan installment day	61(2.23)
Others*[1]	39(1.43)
* refers multiple responses<br>*[1] refers to recall child's date of birth, to calculate the lunar date, etc.	

Calendar literacy and its associated factors

Association between calendar literacy and socio-demographic characteristics

Supplementary Table 1 indicates the majority participants (67%) from age groups 20-29 were calendar literate. The employed women (81%) had more calendar literacy than the unemployed women (62%). Most of the women (89%) who completed eight or more years of schooling were calendar literate. Ninety percent of the women who did not complete five years of schooling were calendar illiterate.

Educational status of calendar literate women

In Figure 2, we explored the relationship between calendar literacy and the educational background of the study participants. Seventy-two percent of women who completed eight or more years of schooling could mark the correct date in the calendar. However, only a few of the women (3%) who did not complete five years of schooling could also mark the calendar correctly.

For peer review only - http://bmjopen.bmj.com/site/about/guidelines.xhtml

Factors associated with calendar literacy

Supplementary Table 2 presents that almost all the indicators were crudely associated with a higher prevalence of calendar literate women. After adjustment, the odds ratio of calendar literate women who completed eight or more years of schooling was 34 times (adj. OR 34.46, 95% CI: 23.96, 49.57) higher than the women who did not complete five years of schooling. The women who belonged to the top two wealth quintiles (i.e. rich adj. OR 2.75, 95% CI: 1.83, 4.11, and richest adj. OR 3.15, 95% CI: 1.88, 5.29) were almost 3 times more calendar literate than the poorer women.

LMP recall

Table 3 presents more than half of the participants (53%) could recall their recent LMP dates. About 59% of women aged ≤19 years recalled their LMP dates. We found a significant statistical (p<0.01) association between the completed years of schooling and the recent LMP recall. The women who had completed eight or more years of schooling, most of them (60%) could recall the LMP date. The women who were calendar literate, about 60% of them could recall their LMPs, and there was a significant (p-value<0.01) association between the calendar literacy and the recent LMP recall.

Table 3 Association between LMP recall and socio-demographic characteristics

Traits	Recall recent LMP		P-value
	**Yes 1446 (%)**	**No 1285 (%)**	
**Age in years**			
≤19	359(58.95)	250(41.05)	
20-29	899(53.10)	794(46.90)	<0.01
30+	188(43.82)	241(56.18)	
**Completed years of schooling**			
<5	201(37.15)	340(62.85)	
5-7	406(51.01)	390(48.99)	<0.01
≥ 8	839(60.19)	555(39.81)	
**Residence**			

For peer review only - http://bmjopen.bmj.com/site/about/guidelines.xhtml

Rural	948(52.20)	868(47.80)	<0.01
Urban	498(54.43)	417(45.57)	
**Employment status of the participant**			
Unemployed	1365(52.85)	1218(47.15)	<0.01
Employed*1	81(54.73)	67(45.27)	
**Gravida**			
1	669(64.45)	369(35.55)	<0.01
≥2	777(45.89)	916(54.11)	
**Wealth index**			
Poorer	236(43.14)	311(56.86)	<0.01
Poor	287(52.56)	259(47.44)	
Middle	294(53.85)	252(46.15)	
Rich	307(56.23)	239(43.77)	
Richest	322(58.97)	224(41.03)	
**Availability of calendar at home**			
Yes	893(56.02)	701(43.98)	<0.01
No	553(48.64)	584(51.36)	
**Purpose of using calendar*2**			
To calculate the days and dates	703(58.39)	501(41.61)	0.04
To track the dates of the menstrual period	168(62.69)	100(37.31)	
To remember the special/festival day (Hat day/ Eid/Puja/Selling certain goods)	288(65.45)	152(34.55)	
To remember the date of the tuition fee of children	55(57.89)	40(42.11)	
To remember the loan installment day	33(54.10)	28(45.90)	
Others*3	22(56.41)	17(43.59)	
**Calendar literacy**			
Literate	1034(59.77)	696(40.23)	<0.01
Illiterate	412(41.16)	589(58.84)	
**Track LMP date**			
Written	1394(53.86)	1194(46.14)	<0.01
Unwritten	52(36.36)	91(63.64)	

Total	1446 (52.95)	1285 (47.05)	
*1 refers to Service/Business/Handicraft/Agriculture/Farm/Fishing, and Day labour, etc.<br>*2 refers to multiple responses<br>*3 refers to recall child's date of birth, to calculate the lunar date, etc.			

LMP recall and its associated factors

Table 4 present that almost all the indicators were crudely associated with the higher prevalence of recalling LMP. After adjustment, the odds ratio of LMP recall for the women who completed eight or more years of schooling was 1.39 times (adj. OR 1.39, 95% CI: 1.05, 1.84) higher than the women who did not complete five years of schooling. Similarly, the richest women were more likely to recall recent LMP than the poorer women (rich: adj. OR 1.46, 95% CI: 1.08, 1.97 and richest: 1.55, 95% CI: 1.09, 2.21). Besides, the women who had primigravida were more likely to recall recent LMP than those who had multigravida (adj. OR 1.88, 95% CI: 1.55, 2.29). The odds ratio of LMP recall for calendar literate women was 1.59 times (adj. OR 1.59, 95% CI: 1.28, 1.98) higher than the calendar illiterate women.

Table 4 Crude and adjusted OR for the factors associated with LMP recall

Covariates	cOR (95% CI lower to upper)	aOR	(95% CI lower to upper)	P-value
**Constant**		0.41	(0.31 to 0.55)	<0.01*
**The age group of women**				
≤19 years	1.84 (1.43 to 2.36)	1.10	(0.81 to 1.49)	0.53
20 to 29 years	1.45 (1.17 to 1.80)	1.10	(0.88 to 1.38)	0.42
≥ 30 years	1.0	1.0		
**Completed years of schooling**				
<5	1.0	1.0		
5-7	1.76 (1.41 to 2.20)	1.26	(0.98 to 1.61)	0.07
≥8	2.56 (2.08 to 3.14)	1.39	(1.05 to 1.84)	0.02*
**Residence**				
Rural	1.0	1.0		

For peer review only - http://bmjopen.bmj.com/site/about/guidelines.xhtml

Urban	1.09 (0.93 to 1.28)	0.81	(0.64 to 1.02)	0.07
**Employment status of the participant**				
Unemployed	1.0	1.0		
Employed*	1.08 (0.77 to 1.50)	0.85	(0.60 to 1.21)	0.37
**Gravida**				
1	2.14 (1.82 to 2.51)	1.88	(1.55 to 2.29)	<0.01*
≥2	1.0	1.0		
**Wealth index**				
Poorer	1.0	1.0		
Poor	1.46 (1.15 to 1.85)	1.26	(0.98 to 1.62)	0.06
Middle	1.54 (1.21 to 1.95)	1.28	(0.99 to 1.66)	0.06
Rich	1.69 (1.33 to 2.15)	1.46	(1.08 to 1.97)	0.01*
Richest	1. 89 (1.49 to 2.41)	1.55	(1.09 to 2.21)	0.01*
**Availability of calendar at home**				
No	1.0	1.0		
Yes	1.35 (1.15 to 1.57)	0.94	(0.73 to 1.22)	0.65
**Using calendar to calculate days and dates**				
No	1.0	1.0		
Yes	1.48 (1.27 to 1.72)	0.94	(0.73 to 1.23)	0.67
**Using a calendar to track the dates of the menstrual period**				
No	1.0	1.0		
Yes	1.58 (1.22 to 2.06)	1.25	(0.94 to 1.65)	0.12
**Calendar literacy**				
Literate	2.12 (1.81 to 2.49)	1.59	(1.28 to 1.98)	<0.01*
Illiterate	1.0	1.0		

Significant p-value ≤0.05.
aOR, adjusted OR; cOR, crude OR.
CI – Confidence interval

For peer review only - http://bmjopen.bmj.com/site/about/guidelines.xhtml

*refers to Service/Business/Handicraft/Agriculture/Farm/Fishing, and Day labour, etc.
The model adjusted for variables such as woman's age, completed years of schooling, residence, employment status, gravida, wealth index, availability of calendar at home, the purpose of using a calendar, tracking LMP, and calendar literacy.

Discussion

This study explored the prevalence of LMP recall and calendar literacy along with the factors associated with these two outcome variables. This study suggested, urban women were more calendar literate than their rural counterparts but we did not find any significant difference between these two groups on the prevalence of recalling recent LMP. This study also found, more than half of the women had calendars available at their home, however, only a few women used the calendar to track their LMPs. As expected, calendar literate women could better recall their LMPs than the calendar illiterate women. Besides, years of schooling, fewer pregnancies, and socioeconomic class were the significant associated factors for recalling LMP. Women with eight or more years of schooling, first time pregnancy, and women from the richest wealth quintile could recall their LMP better.

Because of the unavailability of the studies reporting the prevalence of calendar literacy and the factors associated with calendar literacy, and how calendar literacy contributes to LMP recall we could not compare our findings with any study. We could only compare our findings on the prevalence of LMP recall. However, we discussed the use and importance of calendar method or LMP recall in relation to findings from prior studies around the globe including Bangladesh.

Similar to our findings, a study conducted in 2005 in a developed country found that more than half of the women were capable to recall their LMP accurately.[18] A study conducted in Bangladesh in 2016 examined the significance of LMP recall to measure the gestational age by using the home calendar and explored how it differed from the ultrasound result. The study validated the use of the LMP method for estimating gestational age in a rural setting of Bangladesh. The same study also revealed that a mother's higher education level and previous experience of preterm birth is associated with better accuracy of LMP recall.[35] Studies also evidenced that LMP is a reliable way

For peer review only - http://bmjopen.bmj.com/site/about/guidelines.xhtml

to calculate the gestational age while one study had the precision level of 86-90% for LMP recall's accuracy and reliability.[13 36]

Several studies prioritised the LMP method to determine the gestational age as well as to assess the preterm birth and fetal growth restriction. [14 37-39] A number of studies used the LMP recall method to identify pregnancy and utilised paper-based calendar as a tool to get the accurate LMP recall and recruited pregnant women immediately after pregnancy confirmation for their surveillance or interventions.[12-14 18 35-43] Our cross-sectional survey only explored the use of a calendar, calendar literacy, LMP recall and its associated factors in the area where there is no such paper-based calendar intervention. In our country context, women usually remember LMP dates in an unwritten way. This study found that only 10% of women used a calendar for tracking LMP. However, in the modern era, everyone seems to be connected to the digital world, with more and more people using calendars inbuilt in their phone, their email, or various other options. Many places like homes and businesses still use at least one printed paper calendar.[44] This study also found that 58% of households owned a printed calendar and 93% owned (according to our household asset calculation) at least one cell phone. The printed calendar provides an easy way to see the date without having to log in and access a tiny screen on the smartphone. So, using a printed calendar to track LMP is still comfortable and relevant for women even in the age of digital technology where an electronic calendar is available on the cell phone.

However, the LMP method has its limitations. LMP can predict gestational age accurately only if the cycle characteristics and the date of onset of the last menstrual bleed can be established.[36 40] Despite the limitations, studies conducted in different geographical settings such as Bangladesh and Guatemala have recommended LMP to be the chosen method for determining gestational age in low-resource settings.[12 41 43]

Since our study was conducted in the Northern part of Bangladesh, where ANC coverage is higher, the literacy rate is moderate, and human resource gaps are low; hence, the findings are subject to compare contextually. The analysis was done depending on self-reported information without having a strong surveillance system; therefore, the scope of over or under-reporting may exist. Because of the self-reported data, LMP recall may vary.

For peer review only - http://bmjopen.bmj.com/site/about/guidelines.xhtml

There may be some other confounding factors such as lack of awareness about the importance of gestational age, and irregular menstrual cycle were not explored in this study. In Bangladesh, tracking one's menstrual cycle using a calendar is a concept that is yet to become familiar and practised. If women are calendar literate, they will be able to have a clear idea about their menstrual cycle, monitor, and detect any menstrual abnormality. In addition to these, they will be able to calculate the gestational age properly and identify pregnancy at an earlier stage. Early identification of pregnancy can lead to proper and timely utilisation of ANC. This would prevent complications during pregnancy and delivery for the mothers and the babies, thus to improve birth outcomes. We, therefore, feel a high necessity of further nationwide research on this very topic to improve maternal and child health and smoothing the path to achieve Sustainable Development Goals.

Conclusion and recommendation

Accurate recalling of LMP is crucial for maternal health as it helps to calculate the gestational age accurately. It also helps to detect pregnancy early which can influence early initiation of pregnancy care. We found a significant relationship between calendar literacy and LMP recall. Though the calendar literacy rate and the availability of the calendar at home are reasonable, however; the practice of LMP tracking using a calendar is shallow. Besides calendar literacy, factors like years of schooling, place of residence, and socio-economic status have a significant influence on LMP recall too. Mounting all the relevant evidence, we summed up that calendar literacy is an opportunity to introduce a simple tool like paper-based calendar in any Maternal, Neonatal, and Child Health programmes at communities. And this would be very useful, especially where ultrasound is not available to ensure accurate LMP recall for early pregnancy registration and timely ANC coverage for improving maternal health outcomes in Bangladesh.

Authors' contributions

BKS, MR, DKM, MKM, AA and AR conceived and designed the experiments. BKS, MR, TR2, JJK, MH and MSM performed the experiments. BKS, TR1, TR2, FA, MH, SNM, AA and AR were involved in analysing data. BKS, MR, TR2, JJK, TR1, MH, FA, MSM and FR contributed

For peer review only - http://bmjopen.bmj.com/site/about/guidelines.xhtml

to developing the tools. BKS, MR, TR1, TR2, FR, SNM, DKM, MKM, AA and AR wrote the paper.

Competing interests

The authors declare that they have no competing interests.

Funding statement

This research protocol was funded by the Bill & Melinda Gates Foundation. The awarded BMGF grant number is OPP1146943.

Data availability statement

Data are available upon reasonable request. Unpublished data are available in accordance with icddr,b's data sharing policy. Requests should be addressed to Ms. Armana Ahmend, Head, Research Administration, icddr,b; aahmed@icddrb.org. More information can be found here- https://eur01.safelinks.protection.outlook.com/?url=https%3A%2F%2Fwww.icddrb.org%2Fdmdocuments%2Ficddrb%2520Data%2520Access%2520Policy.pdf&data=02%7C01%7Canne.laterra%40care.org%7C73604d2a11bc4750c21608d75e12da16%7Ce83233b748134ff5893ff60f400bfcba%7C0%7C1%7C637081307376649360&sdata=fLeMIOsL2d%2FnkdoDykAoa9kmPxhn3hnJutjwEkOScI4%3D&reserved=0.

Patient and Public Involvement

Patients or the public were not involved in the design, or conduct, or reporting, or dissemination plans of this study

Acknowledgement

This research protocol was funded by the Bill & Melinda Gates Foundation. The awarded BMGF grant number is OPP1146943. icddr,b acknowledges with gratitude the commitment of Bill & Melinda Gates Foundation to its research efforts. We are grateful to our study participants for their spontaneous participation and sincere commitment to fulfil the research endeavour. icddr,b

For peer review only - http://bmjopen.bmj.com/site/about/guidelines.xhtml

is also grateful to the Governments of Bangladesh, Canada, Sweden and the UK for providing core/ unrestricted support.

Figure legends

"Figure 1: Women who could mention current date."

"Figure 2: Educational status of calendar literate women."

For peer review only

References

1. Alkema L, Chou D, Hogan D, et al. Global, regional, and national levels and trends in maternal mortality between 1990 and 2015, with scenario-based projections to 2030: a systematic analysis by the UN Maternal Mortality Estimation Inter-Agency Group. *The Lancet* 2016;387(10017):462-74.
2. Wanjira C, Mwangi M, Mathenge E, et al. Delivery practices and associated factors among mothers seeking child welfare services in selected health facilities in Nyandarua South District, Kenya. *BMC public health* 2011;11(1):360.
3. Streatfield PK, El Arifeen S, Al-Sabir A, et al. Bangladesh maternal mortality and health care survey 2010: summary of key findings and implications. *Dhaka, Bangladesh: National Institute of Population Research and Training (NIPORT)* 2011
4. National Institute of Population Research Training , International Centre for Diarrhoeal Disease Research Bangladesh , MEASURE Evaluation. Bangladesh Maternal Mortality and Health Care Survey 2016: Preliminary Report: NIPORT, icddr, b, and MEASURE Evaluation Dhaka, Bangladesh, and Chapel Hill, 2017.
5. Dunson DB, Colombo B, Baird DD. Changes with age in the level and duration of fertility in the menstrual cycle. *Human reproduction* 2002;17(5):1399-403.
6. Sohda S, Suzuki K, Igari I. Relationship between the menstrual cycle and timing of ovulation revealed by new protocols: analysis of data from a self-tracking health app. *Journal of medical Internet research* 2017;19(11):e391.
7. Stanford JB, White Jr GL, Hatasaka H. Timing intercourse to achieve pregnancy: current evidence. *Obstetrics & Gynecology* 2002;100(6):1333-41.

For peer review only - http://bmjopen.bmj.com/site/about/guidelines.xhtml

8. Wilcox AJ, Weinberg CR, Baird DD. Timing of sexual intercourse in relation to ovulation—effects on the probability of conception, survival of the pregnancy, and sex of the baby. *New England Journal of Medicine* 1995;333(23):1517-21.

9. Tunon K, Eik-Nes S, Grøttum P. A comparison between ultrasound and a reliable last menstrual period as predictors of the day of delivery in 15 000 examinations. *Ultrasound in Obstetrics and Gynecology: The Official Journal of the International Society of Ultrasound in Obstetrics and Gynecology* 1996;8(3):178-85.

10. Ghani A, Nahar A, Sultana N, et al. Prediction of Gestational age by Last Menstrual Period (LMP) in Comparison to Ultrasonography (USG). *Journal of Shaheed Suhrawardy Medical College* 2014;6(2):82-86.

11. World Health Organization. Home-based maternal records: guidelines for development, adaptation and evaluation: World Health Organization 1994.

12. Salam SS, Ali NB, Rahman AE, et al. Study protocol of a 4-parallel arm, superiority, community based cluster randomized controlled trial comparing paper and e-platform based interventions to improve accuracy of recall of last menstrual period (LMP) dates in rural Bangladesh. *BMC public health* 2018;18(1):1-8.

13. Rosenberg RE, Ahmed ANU, Ahmed S, et al. Determining gestational age in a low-resource setting: validity of last menstrual period. *Journal of health, population, and nutrition* 2009;27(3):332.

14. Karl S, Suen CSLW, Unger HW, et al. Preterm or not–an evaluation of estimates of gestational age in a cohort of women from rural Papua New Guinea. *PloS one* 2015;10(5):e0124286.

15. Macaulay S, Buchmann EJ, Dunger DB, et al. Reliability and validity of last menstrual period for gestational age estimation in a low-to-middle-income setting. *Journal of Obstetrics and Gynaecology Research* 2019;45(1):217-25.

16. Van Oppenraaij R, Eilers P, Willemsen S, et al. Determinants of number-specific recall error of last menstrual period: a retrospective cohort study. *BJOG: An International Journal of Obstetrics & Gynaecology* 2015;122(6):835-41.

17. Waller DK, Spears W, Gu Y, et al. Assessing number-specific error in the recall of onset of last menstrual period. *Paediatric and Perinatal Epidemiology* 2000;14(3):263-67.

18. Wegienka G, Baird DD. A comparison of recalled date of last menstrual period with prospectively recorded dates. *Journal of Women's Health* 2005;14(3):248-52.

For peer review only - http://bmjopen.bmj.com/site/about/guidelines.xhtml

19. Kamana K, Shakya S, Zhang H. Gestational diabetes mellitus and macrosomia: a literature review. *Annals of Nutrition and Metabolism* 2015;66(Suppl. 2):14-20.

20. Cnattingius S, Villamor E, Lagerros Y, et al. High birth weight and obesity—a vicious circle across generations. *International journal of obesity* 2012;36(10):1320-24.

21. Salahuddin M. Predictors of severe obesity in low-income, predominantly Hispanic/Latino children: the Texas Childhood Obesity Research Demonstration Study. *Preventing chronic disease* 2017;14

22. Poma PA. Early detection of pregnancy. *Journal of the National Medical Association* 1984;76(3):305.

23. Bangladesh Bureau of Statistics, Statistics and Informatics Division, Ministry of Planning. Bangladesh Population and Housing Census 2011, Community report, Dinajpur: Bangladesh Bureau of Statistics (BBS), Statistics and Informatics Division (SID), Ministry of Planning, 2014.

24. Bangladesh Bureau of Statistics, Statistics and Informatics Division, Ministry of Planning. Population and Housing Census, Community report, Nilphamari: Bangladesh Bureau of Statistics (BBS), Statistics and Informatics Division (SID), Ministry of Planning, 2013.

25. Bangladesh Bureau of Statistics, Statistics and Informatics Division, Ministry of Planning. District Statistics 2011, Rajshahi: Bangladesh Bureau of Statistics (BBS), Statistics and Informatics Division (SID), Ministry of Planning, 2013.

26. Bangladesh Bureau of Statistics, Statistics and Informatics Division, Ministry of Planning. Population & Housing Census 2011: Bangladesh Bureau of Statistics (BBS), Statistics and Informatics Division (SID) and Ministry of Planning, 2015.

27. MEASURE Evaluation, ICDDR B. Bangladesh District level socio-demographic and health care utilization indicators: National Institute of Population Research and Training (NIPORT), MEASURE Evaluation, UNC-CH, USA and ICDDR,B, 2011.

28. Human Resource management, Ministry of Health and Family Welfare. HRH Data Sheet 2014: Human Resource management (HRM), Ministry of Health and Family Welfare (MOHFW), Bangladesh Secretariat, Dhaka, 2014.

29. Hayes R, Bennett S. Simple sample size calculation for cluster-randomized trials. *International journal of epidemiology* 1999;28(2):319-26.

For peer review only

For peer review only - http://bmjopen.bmj.com/site/about/guidelines.xhtml

30. Bostoen K, Chalabi Z. Optimization of household survey sampling without sample frames. *International journal of epidemiology* 2006;35(3):751-55.
31. Myatt M. A short guide to undertaking surveys using the Simple Spatial Survey Method (S3M)2012.
32. Vyass S, Kumaranayake L. Constructing socioeconomic status indexes: how to use principal component analysis. *Health Policy Plan* 2006;21(6):459-68.
33. Wold S, Esbensen K, Geladi P. Principal component analysis. *Chemometrics and intelligent laboratory systems* 1987;2(1-3):37-52.
34. United States Agency for International Development (USAID). The DHS Program 2020 [Available from: https://dhsprogram.com/topics/wealth-index/ accessed 26/09/2020.
35. Shah R, Mullany LC, Darmstadt GL, et al. Incidence and risk factors of preterm birth in a rural Bangladeshi cohort. *BMC pediatrics* 2014;14(1):112.
36. Ellertson C, Elul B, Ambardekar S, et al. Accuracy of assessment of pregnancy duration by women seeking early abortions. *The Lancet* 2000;355(9207):877-81.
37. Rashid H, Ma E, Ferdous F, et al. First-trimester fetal growth restriction and the occurrence of miscarriage in rural Bangladesh: A prospective cohort study. *PloS one* 2017;12(7):e0181967.
38. Baqui A, Rosen H, Lee A, et al. Preterm birth and neonatal mortality in a rural Bangladeshi cohort: implications for health programs. *Journal of Perinatology* 2013;33(12):977-81.
39. Lee AC, Mullany LC, Ladhani K, et al. Validity of newborn clinical assessment to determine gestational age in Bangladesh. *Pediatrics* 2016;138(1):e20153303.
40. Unger H, Thriemer K, Ley B, et al. The assessment of gestational age: a comparison of different methods from a malaria pregnancy cohort in sub-Saharan Africa. *BMC pregnancy and childbirth* 2019;19(1):1-9.
41. Persson LÅ, Arifeen S, Ekström E-C, et al. Effects of prenatal micronutrient and early food supplementation on maternal hemoglobin, birth weight, and infant mortality among children in Bangladesh: the MINIMat randomized trial. *Jama* 2012;307(19):2050-59.
42. Gernand AD, Paul RR, Ullah B, et al. A home calendar and recall method of last menstrual period for estimating gestational age in rural Bangladesh: a validation study. *Journal of Health, Population and Nutrition* 2016;35(1):34.

For peer review only - http://bmjopen.bmj.com/site/about/guidelines.xhtml

43. Neufeld LM, Haas JD, Grajéda R, et al. Last menstrual period provides the best estimate of gestation length for women in rural Guatemala. *Paediatric and perinatal epidemiology* 2006;20(4):290-98.

44. VIIKING. Why Printed Calendars are Still Relevant in the Digital Age [cited 2020 08/07/2020]. Available from: https://mrshirt.com/printed-calendars-are-still-relevant.

For peer review only

For peer review only - http://bmjopen.bmj.com/site/about/guidelines.xhtml

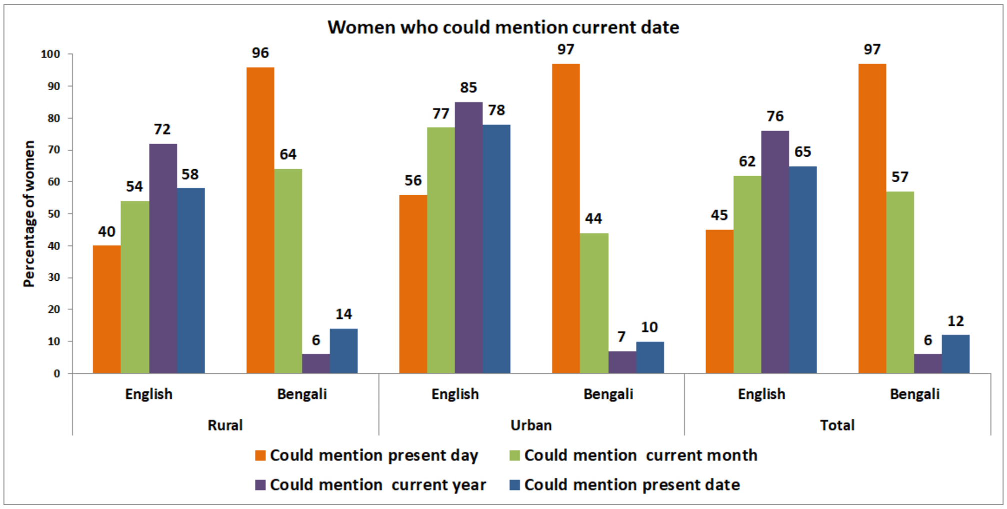

Figure 1: Women who could mention current date

48x24mm (600 x 600 DPI)

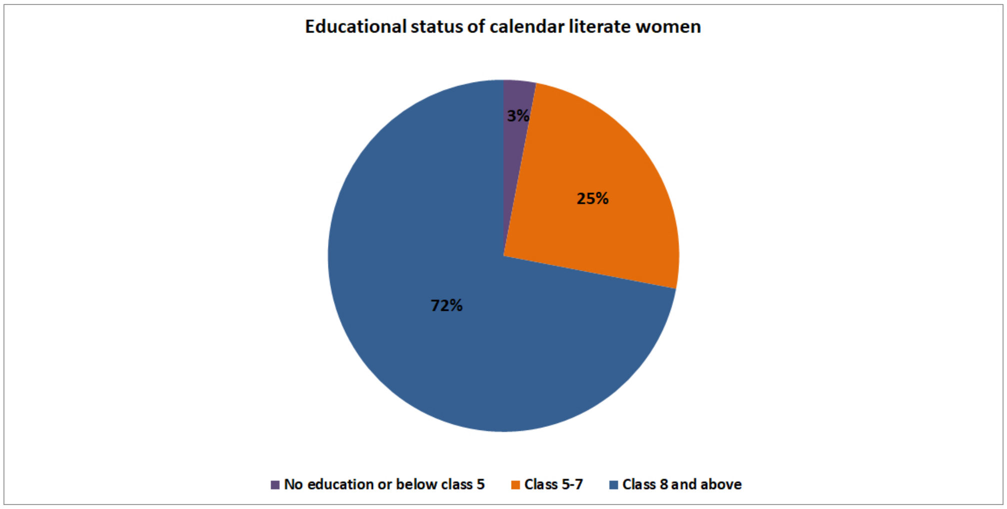

Figure 2: Educational status of calendar literate women

48x24mm (600 x 600 DPI)

Supplementary Table 1 Association between calendar literacy and socio-demographic characteristics of the study participants

Traits	Calendar literacy		P-value
	Literate 1730 (%)	Illiterate 1001 (%)	
**Age in years**			
≤19	362(59.44)	247 (40.56)	<0.01
20-29	1135(67.04)	558 (32.96)	
≥30	233(54.31)	196 (45.69)	
**Completed years of schooling**			
<5	52 (9.61)	489 (90.39)	<0.01
5-7	437 (54.90)	359 (45.10)	
≥ 8	1241 (89.02)	153 (10.98)	
**Residence**			
Rural	1,029 (56.66)	787 (43.34)	<0.01
Urban	701 (76.61)	214 (23.39)	
**Employment status of the participant**			
Unemployed	1610 (62.33)	973 (37.67)	<0.01
Employed*1	120 (81.08)	28 (18.92)	
**Gravida**			
1	751 (72.35)	287 (27.65)	<0.01
≥2	979 (57.83)	714 (42.17)	
**Wealth index**			
Poorer	192(35.10)	355(64.90)	<0.01
Poor	295(54.03)	251(45.97)	
Middle	333(60.99)	213(39.01)	
Rich	423(77.47)	123(22.53)	
Richest	487(89.19)	59(10.81)	
**Availability of calendar at home**			
Yes	1244(78.04)	350(21.96)	<0.01
No	486(42.74)	651(57.26)	

**Purpose of using calendar*2**			
To calculate the days and dates	1085(90.12)	119(9.88)	
To track the dates of menstrual period	241(89.93)	27(10.07)	
To remember the special/festival day (Hat day/Eid/Puja/selling certain goods)	342(93.96)	22(6.04)	<0.01
To remember the date of tuition fee of children	77(90.59)	8(9.41)	
To remember the loan installment day	57(93.44)	4(6.56)	
Others*3	36(92.31)	3(7.69)	

*1 refers Service/Business/Handicraft/Agriculture/Farm/Fishing, and Day labor, etc.

*2 refers multiple responses

*3 refers child's date of birth, calculate lunar date, etc.

Supplementary Table 2 Results from logistics regression on calendar literacy

**Covariates**	cOR (95% CI lower to upper)	aOR	(95% CI lower to upper)	P value
**Constant**		0.05	(0.03 to 0.08)	<0.01*
**Age in years**				
≤19 years	1.23 (0.96 to 1.58)	0.95	(0.61 to 1.47)	0.81
20 to 29 years	1.71 (1.38 to 2.12)	1.20	(0.84 to 1.69)	0.31
≥ 30 years	1.0	1.0		
**Completed years of schooling**				
<5	1.0	1.0		
5-7	11.4 (8.33 to 15.73)	8.35	(5.94 to 11.73)	<0.01*
≥8	76.27 (54.75 to 106.26)	34.46	(23.96 to 49.57)	<0.01*
**Residence**				
Rural	1.0	1.0		
Urban	2.51 (2.09 to 2.99)	1.20	(0.85 to 1.69)	0.30

**Employment status of the participant**				
Unemployed	1.0	1.0		
Employed*	2.59 (1.70 to 3.94)	1.83	(0.99 to 3.38)	0.05
**Gravida**				
1	1.91 (1.61 to 2.25)	1.21	(0.91 to 1.60)	0.18
≥2	1.0	1.0		
**Wealth index**				
Poorer	1.0	1.0		
Poor	2.17 (1.70 to 2.77)	1.71	(1.25 to 2.34)	<0.01*
Middle	2.89 (2.26 to 3.69)	1.28	(0.92 to 1.77)	0.139
Rich	6.36 (4.87 to 8.30)	2.75	(1.83 to 4.11)	<0.01*
Richest	15.26 (11.06 to 21.06)	3.15	(1.88 to 5.29)	<0.01*
**Availability of calendar at home**				
No	1.0	1.0		
Yes	4.76 (4.03 to 5.63)	0.65	(0.48 to 0.88)	<0.01*
**Purpose of using calendar**				
To calculate the days and dates/ installation day	12.47 (10.06 to 15.46)	6.41	(4.57 to 8.98)	<0.01*
To track the dates of menstrual period	5.84 (3.89 to 8.76)	1.08	(0.66 to 1.76)	0.76

Significant p value ≤0.05.
aOR, adjusted OR; cOR, crude OR.
CI – Confidence interval
*****refers Service/Business/Handicraft/Agriculture/Farm/Fishing, and Day labor, etc.
Model adjusted for variables such as woman's age, completed years of schooling, residence, employment status, gravida, wealth index, availability of calendar at home, purpose of using calendar

Research Checklist

Section/Topic	Item #	Description	Reported on page #
**Title and abstract**	1	(*a*) Study design within abstract	2
		(*b*) Summary of what was done and what was found	2
**Strength and Limitation**	2	Four bullet points on strength and limitation of this Study	3
**Introduction**			
Background/rationale	3	Scientific background and rationale of this study	4-5
Objectives	4	Specific objectives of this study	5
**Materials and Methods**			
Study design and settings	5	Study design, settings of the study, data collection period	5-6
Sampling and study participants	6	Sampling strategy, sample size, inclusion criteria of study participants	6-7
Data collection	7	Survey tool development, staff recruitment and training	7-8
Data management and quality assurance	8	How the quality of data was ensured, how the data were managed	8
Ethics	9	How the research integrity and ethics ensured during the study	8-9
Variables and Measures	10	Primary and secondary outcome variables, Operational definition of primary, secondary and other independent variable	10-11
Statistical analysis	11	Descriptive statistics, multiple logistic regression, Principal Component Analysis are done for the statistical analysis	11
**Results**			
Socio-demographic characteristics of the study participants	12	Age, completed years of schooling, place of residence, employment status and wealth index of the study participants are presented	12-13
Descriptive data	13	Characteristics of study participants (e.g. Age, completed years of schooling, place of residence, employment status and wealth index)	12-13
Calendar literacy	14	Prevalence of calendar literacy of the study participants is presented	13-14
Educational status of calendar literate women	15	Distribution of educational status among calendar literate women is presented	14
Calendar literacy and its associated factors	16	Association between calendar literacy and socio-demographic characteristics	14
LMP recall	17	Prevalence of LMP recall of the study participants	15-19
Main results	18	(a) Association between LMP recall and socio-demographic characteristics	15-17
		(*b*) Crude and adjusted OR for the factors associated with LMP recall with the precision of 95% confidence interval. Covariates such as: age, educational status, place of residence, occupation and wealth index were adjusted	17-19
**Discussion**			
Key results	19	Summary of key results	19
Limitations	20	Limitations of the study is described	21
Interpretation	21	Overall interpretation of results considering objectives, limitations, multiplicity of analyses, results from similar studies, and other relevant evidence	19-21
Generalisability	22	Discussion about generalisability of the study results	21
**Other information**			

For peer review only

Funding	23	Funding statement of the study	22
Authors' contribution and acknowledgement	24	Authors' contribution and acknowledgement of the study described	22
Reference	25	References used for this study	23-27

For peer review only
